# A Whodunit Gamified Flipped Classroom For High Yield Bite Injuries And Envenomation

**DOI:** 10.21980/J88S81

**Published:** 2024-10-31

**Authors:** Mary G McGoldrick, Laryssa Patti, Meigra Chin, Tiffany Murano

**Affiliations:** * Columbia University Vagelos College of Physicians & Surgeons, Department of Emergency Medicine, New York, NY; ^ Rutgers Robert Wood Johnson Medical School, Department of Emergency Medicine, New Brunswick, NJ

## Abstract

**Audience:**

Clerkship-level medical students, sub-interns, junior and senior residents, attending physicians

**Introduction:**

Bite injuries and envenomation are core content found in the model of the clinical practice of emergency medicine.^1^ However, depending on the geographic location of training or clinical practice, physicians may or may not be exposed to these pathologies. For example, a qualitative analysis conducted in 2022 discovered a significant range in emergency medicine (EM) physician perception of snake antivenom use and level of comfort, noting that experiences with its use ranged from hundreds of cases treated to purely didactic understanding.^2^ Such discrepancies necessitate supplemental education and activities to bridge the knowledge gap. Ideally, these activities would utilize tenets of experiential learning to allow learner processing comparable to that of clinical experience.^3^ Flipped classroom and audience participation promote engagement and active learning when compared to the passive learning of lectures.^4^ In that vein, there is a growing body of gamified resources in medical education which utilize pattern recognition and problem solving skills that can be analogous to clinical practice.^5^,^6^

**Educational Objectives:**

By the end of this activity, learners will be able to: 1) identify and name species responsible for bite/sting/envenomation injuries, 2) recognize associated signs, symptoms, physical exam findings and complications associated with bites/stings/envenomations by certain species, 3) discuss management such as antibiotics, antivenom, and supportive care.

**Educational Methods:**

We designed a small group activity asking residents to identify, research, and present the “culprits” implicated in environmental exposures to animals and insects, and match them to corresponding clinical scenarios.

**Research Methods:**

Participants anonymously answered electronic multiple-choice quizzes before and after completing the activity to gauge its effectiveness in conveying the material. They also completed an additional anonymous, electronic survey regarding their attitudes towards this activity and the possibility of other gamified didactics within the curriculum.

**Results:**

Each resident class showed an upward trend in their average multiple-choice score, the greatest of which was seen in the post-graduate year (PGY) 1 class. The residency demonstrated a statistically significant improvement in their ability to answer multiple choice questions (MCQs), with an average pre-activity score of 67.14%, and post-activity score of 87.14%. Participants showed determination and enthusiasm to engage with the material when presented in a gamified format, and 100% of post-activity survey respondents wanted to participate in further gamified activities.

**Discussion:**

Gamified small group activities are a fun and effective method of supplementing residency and medical student education for both common and esoteric clinical presentations that they may not encounter in the clinical environment.

**Topics:**

Toxicology, bite injury, envenomation, gamification.

## USER GUIDE

[Table t1-jetem-9-4-sg13]

**Table t1-jetem-9-4-sg13:** 

List of Resources:	
Abstract	13
User Guide	15
Small Groups Learning Materials	20
Appendix A: Pre- and Post-Activity Multiple Choice Questions	20
Appendix B: Suspects and Clinical Scenarios PowerPoint Slides	21
Appendix C: sGAE Answers	22
Appendix D: Post-Activity Survey	23

**Learner Audience:**

Medical Students, Interns, Junior Residents, Senior Residents, Attendings

**Time Required for Implementation:**

Pre-reading: asynchronous, 30 minutes

Pre-activity multiple choice questions: 5 minutes, optional

Activity content: 40 minutes

- 15 minutes small-group, independent research of “suspect(s)”
- 25 minutes learner identification of correct scenario and small group teach-back/presentation of corresponding “suspect”

Post-activity multiple choice questions, survey (optional): 8 minutes

Distribution of summary “pearls”: 2 minutes

**Recommended Number of Learners per Instructor**:

Twenty learners per facilitator. Easily administered to larger groups but would recommend additional facilitators available for questions during small group “research” period.

Small group size: 2–3 learners per “suspect,” more if larger group of learners.

**Topics:**

Toxicology, bite injury, envenomation, gamification.

**Objectives:**

By the end of this small group exercise, learners will be able to:

1. Identify and name species responsible for bite and sting/envenomation injuries common to the scope of practice of emergency medicine.
2. Recognize associated signs, symptoms, and physical exam findings.
3. Review possible complications by species (i.e., specific bacteria or toxins).
4. Discuss management such as antibiotics, antivenoms, and supportive care.

### Linked objectives and methods

This small group activity utilized elements of asynchronous learning, the flipped classroom model, and gamified framing to generate a greater depth of interaction with bites, stings, and envenomations than learners would receive in a traditional lecture format.

At the beginning of the environmental didactic block, residents were encouraged to review the free, open-access medical education resource, Foundations of Emergency Medicine (FoEM). Its environmental curriculum contains materials such as blog posts, podcasts, and recommended samples from primary texts.^7^ This was not required, and participants in the small group activity were not screened for its completion.

At the beginning of the session, learners were presented with a series of images (via Microsoft PowerPoint [Appendix B]) of common “suspects” frequently identified as causes of high-yield environmental emergencies in EM and assigned one or more suspects to “research.” In small groups, they were required to identify their assigned animal or insect based on the image that they were shown (objective 1). After establishing the responsible organism, they were then asked to utilize a FOAMed resource of their choice to review typical patient presentations and complications caused by any specific bacteria or toxin associated with the organism (objectives 2 and 3), and to study how each emergency would be managed (objective 4). After the research period, a series of clinical scenario slides were presented. After each scenario, the learners were asked to utilize their knowledge and indicate whether their organism was the “culprit” behind the given scenario. If correct, the responsible small group came to the front of the room to present their “research” to justify their answer and educate the larger group on signs and symptoms, associated complications, and EM management. This was repeated until all nine “suspects” had been matched to a scenario and presented, ensuring each of the learning objectives were achieved for all participants in all nine high-yield organisms. Finally, a “pearls” sheet summarizing all educational content was distributed to each participant to solidify the concepts presented.

### Recommended pre-reading for facilitator

- Donaldson R, Marschall M. Ostermayer D, et al. Mammalian bites, *WikEM*. 2024. Accessed: 15 February 2024. https://wikem.org/wiki/Mammalian_bites
- Donaldson R, Swartz J, Ostermayer D. Envenomatrions, bites and stings. *WikEM*. 2016. Accessed: 15 February 2024. https://wikem.org/wiki/Envenomations,_bites_and_stings
- Schneir A. Clark RF. Bites and stings. In: Tintinalli JE, Ma OJ, Yealy DM, et al, eds. *Tintinalli's Emergency Medicine: A Comprehensive Study Guide*. 9th ed. McGraw Hill; 2020:1053–1062.

### Learner responsible content (LRC)

- Moore KG. Foundations I – Environmental Exposures. Tox/Environmental. Foundations of Emergency Medicine. 2023. https://foundationsem.com/tox-environmental/

### Instructions for facilitators

1. (Optional) Allow 5 minutes for learners to individually complete the multiple-choice questions by having them scan the QR code (slide 2).
2. Learners self-select into nine small groups, containing at least one junior (medical student, PGY-1) and one senior (PGY-2, PGY-3) learner. If cohort size is smaller than 18, a group can be assigned more than one “suspect.”
3. Present the “suspect” slides. Group number one is presented with the slide containing the image of “suspect” number one, followed by group two, etc. until each group has been assigned their suspect.
4. Learners begin the dedicated “research” period where they utilize the FOAMed resource of their choice to elicit the information requested on the instruction slide titled, “The Rules” (slide 12, to be left up during the research period).
5. After 15 minutes of “research,” facilitator should display scenario 1. The learners will be asked which suspect is responsible for the corresponding scenario. One of the small groups responds, and they will present their “evidence” to the rest of the groups. If they are correct, advance to the following scenario. If they are not correct, invite another small group to challenge with their own “evidence.” Repeat until all scenarios are completed and each small group has presented their “case.”
6. Re-iterate correct answers on the “reveal” (slide 22).
7. (Optional) Allow 10 minutes to complete the post-activity MCQs and learner survey (slides 23 and 24).
8. Distribute “Pearls” sheet after activity is completed.

### Results and tips for successful implementation

Our cohort was 14 residents, five PGY-1s, five PGY-2s, and four PGY-3s during weekly didactics, facilitated by one academic faculty member, and one chief resident. Prior to the activity, the residents took five minutes to anonymously complete five multiple choice questions (MCQs) independently (using Google forms questionnaire, Appendix A) on bite and sting scenarios. They submitted their responses electronically and were not provided with the correct answers. After each team matched their suspect to the clinical scenario and all suspects had been presented, the groups were asked to complete the same multiple-choice questions (individually) to ascertain if their scores improved from prior to the activity. Results were analyzed using a two-tailed t-test for significance, examining the pre-activity MCQ scores and comparing them to the post-activity scores by PGY class and as a residency. Each class demonstrated improvement in their ability to answer the questions after completing the activity, the greatest of which was seen in the PGY-1 cohort, but the results were not statistically significant. However, the average pre-activity quiz score for the combined residency was 67.14%. The post-activity quiz average was 87% for the whole residency, a statistically significant improvement (p-value 0.00215). Data was limited by cohort size.

Eight of the 14 participants completed a separate post-activity survey (Appendix D). All respondents answered that they felt the activity improved their knowledge of the topics and would be interested in participating in gamified small-group activities in the future. Individuals commented that they found the game fun and engaging, and that they felt the material was better absorbed when delivered in this format. In its current iteration, our activity achieved Kirkpatrick level 2; learners felt connected to the material (level 1) and demonstrated improved knowledge (level 2).^8^

While our activity utilized a gamified structure to promote fun and encourage participation, it could have contained more competitive elements. For example, there was no clear “winner,” since the goal was for each group to identify the correct scenario-suspect pair while teaching the rest of the participants about their particular “culprit.” This was intended to have the learners use the information to solve the case and then present that information to their peers for an added layer of reinforcement of the content. In future iterations, the activity could be easily modified to promote competition. The authors intend to run the activity again, this time printing each suspect slide from the PowerPoint and distributing them to their assigned group. The scenarios would be printed and displayed throughout the learning space (pinned to white board or placed on tables throughout the room). After designated research time, learners would have to locate and match their suspect card to the correct scenario. The first to match their suspect and scenario would be declared the winner of the activity. Teach-back could occur on several levels: one being interactions with other small groups during the matching period to justify or defend conflicting matches, another being a large group debrief where each team instructs the larger group on their findings.

Additionally, the pre-and post-activity MCQs are optional, but easily modified into a traditional team-based learning (TBL) individual readiness assessment test (iRAT) and group readiness assessment test (gRAT).

### Pearls

[Table t2-jetem-9-4-sg13] [Table t3-jetem-9-4-sg13] [Table t4-jetem-9-4-sg13] [Table t5-jetem-9-4-sg13] [Table t6-jetem-9-4-sg13]

**Table t2-jetem-9-4-sg13:** 

**Human**
Presentation: Assaults, closed fist vs. teeth (fight bite) *More likely than any other mammalian bite to get infected
Management: irrigation, rule out underlying fracture/foreign body, (Tetanus, Diphtheria, Pertussis) Vaccine Tdap
Complications: joint/tendon injury or infection
Bacteria: Staph/Strep, *Eikenella*, *fusobacterium* etc.
Antibiotics (Abx): amoxicillin and clavulanate potassium (Amox-clav) vs. Clindamycin+ Ciprofloxacin (Clinda + cipro) OR trimethoprim–sulfamethoxazole (TMP-SMX) for prophylaxis and treatment

**Bat**
Presentation: Small, pinpoint bites. Often not overtly visible, high index of suspicion if bat found indoors
Management: Rabies Immune Globulin (Rabies Ig) and vaccination, Tdap
Complications: number one carrier of rabies in the United States

**Table t3-jetem-9-4-sg13:** 

**Cat**
Presentation: bite vs. scratch. Bites tend to be deeper, more needle-like, “inject,” bacteria
Management: irrigation, rule out underlying foreign body, Tdap, should heal by secondary intention
Complications: violation of muscle/tendon, subsequent infection. Cat scratch disease – lymphadenopathy, vesicular, papular lesion, possible encephalitis, hepatic disease, fever, fatigue
Bacteria: *Pasturella, Strep/staph* cat scratch: *Bartonella*
Abx: amox-clav vs. doxy vs. Clinda + cipro OR TMP-SMX for prophylaxis and treatment of bites. Azithromycin for scratch

**Table t4-jetem-9-4-sg13:** 

**Dog**
Presentation: extremities vs. face, can be extensive w/significant cosmetic concerns
Management: irrigation, rule out underlying foreign body, Tdap, closure if indicated, consider surgical consult
Complications: violation of muscle/tendon, subsequent infection.
Bacteria: *Pasturella, Strep/staph*
Abx: amox-clav vs. doxy vs. clinda + cipro OR TMP-SMX for prophylaxis and treatment

**Table t5-jetem-9-4-sg13:** 

**Elapid**
Presentation: Puncture, surrounding erythema, oozing. Numbness, altered mental status (AMS), resp. failure. Sea snakes, cobras (red + yellow, “kill a fellow.”)
Management: wound care, mark edge, pressure dress in field. Complete blood count (CBC), coagulation tests, basic metabolic pantel (BMP), liver function tests (LFTs), creatine phosphokinase (CPK)
Complications: neurotoxic resp failure – venom binds Acetylcholine (Ach) receptors
Antivenom: North American Coral Snake Antivenom (*Micrurus fulvius*) (equine orgin)

**Crotalid**
Presentation: Fang marks, local erythema/tissue necrosis, n/v, fasciculations, coagulopathy. Pit vipers, rattlesnakes, copperheads (triangular shaped heads w/“pits” behind the nostrils)
Management: wound care, mark edge, monitor compartments. CBC, coags, fibrinogen, d-dimer, BMP
Complications: tissue damage, coagulopathy, hypotension
Antivenom: Crotalidae Polyvalent Immune Fab, Crofab. Anavip. Indicated if coagulopathy, swelling moves past marks, pt. unstable

**Black Widow (** ***Lactrodectus*** **)**
Presentation: black spider w/red hourglass. Pinprick with increasing pain followed by cramping, severe abdominal pain, headache, photophobia. Latrotoxin causes massive neurotransmitter release
Management: wound care, NSAID/ice, opioids/benzodiazepines, antivenom if systemic symptoms. CBC, CPK, lactate, urinalysis
Complications: low mortality, can cause afib/myocarditis
Antivenom: risk can often outweigh benefit. Only use if abnormal vitals

**Table t6-jetem-9-4-sg13:** 

**Brown Recluse (** ***Loxosceles*** **)**
Presentation: brown “violin,” on body. painless bite followed by local tissue reaction, can blister, become erythematous, ecchymotic, necrotic. Hyaluronidase and sphingomyelinase mediated.
Management: wound care, Tdap, hemolysis labs if indicated
Complications: rarely nausea, vomiting (n/v), fever, disseminated intravascular coagulation (DIC), rhabdomyolysis

**Scorpion (** ***Centuroides)***
Presentation: most scorpions cause local pain. *Centuroides* =pain, paresthesias, CN palsies (abnormal eye movement) and agitation, flailing extremities, hypersalivation, tachycardia
Management: supportive care w/benzodiazepines, intubation/atropine for extreme resp. symptoms.
Complications: resp. distress from hypersalivation, motor agitation. Usually limited to children.
Antivenom: only stocked in southwest US – Centuroides immune Fab. Indicated for psychomotor or resp. distress.

## Supplementary Information



## Figures and Tables

**Figure f1-jetem-9-4-sg13:**
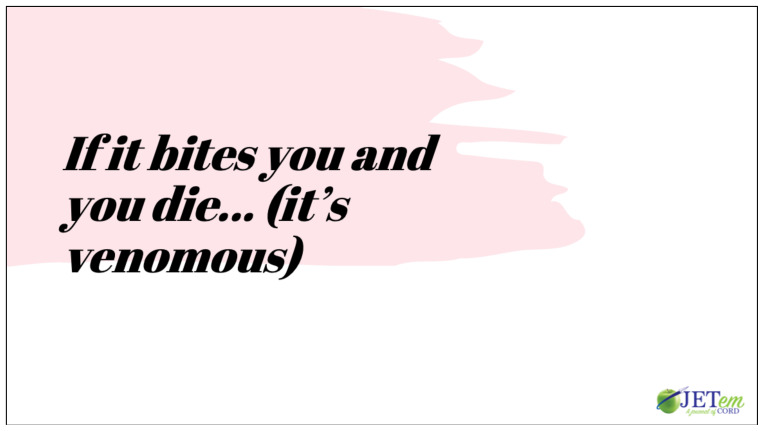
Please see associated PowerPoint file

## SMALL GROUPS LEARNING MATERIALS

### Appendix A Pre- and Post-Activity Multiple Choice Questions

[Table t7-jetem-9-4-sg13]

**Table t7-jetem-9-4-sg13:**

I am a:	PGY-1	PGY-2	PGY-3	PGY-4	Other: ________

1. Bite wounds from which of the following species can result in thrombocytopenia?
  - Black Widow Spider
  - Elapidae (coral snake, cobra)
  - Crotaline (pit vipers)
  - Scorpion
  - Brown Recluse Spider
2. A bite from a _______ is most likely to develop subsequent infection.
  - Human
  - Bat
  - Cat
  - Dog
  - Raccoon
3. A patient presents after being bitten by his newly adopted dog. Which of the following is the most appropriate antibiotic choice?
  - Vancomycin
  - TMP-SMX
  - Amoxicillin-clavulanate
  - Ceftriaxone
  - Doxycycline
4. A patient presents with diaphoresis, tachycardia, myalgias, and abdominal cramping shortly after a mildly painful bite injury. Which of the following caused these symptoms?
  - Black Widow Spider
  - Tarantula
  - Brown Recluse Spider
  - Scorpion
  - Elapidae (coral snake)
5. Which animal is the primary carrier of rabies in the US?
  - Raccoons
  - Foxes
  - Rats
  - Skunks
  - Bats

### Appendix B Suspects and Clinical Scenarios

[Fig f1-jetem-9-4-sg13]

### Appendix C sGAE Answers

Appendix A: Multiple Choice Answers

1. Crotalidae
2. Human
3. Amoxicillin-clavulanate
4. Black Widow Spider
5. Bats

Appendix B: Suspect and Scenario Matches

1. Elapid
2. Brown Recluse Spider
3. Bat (Little Brown Bat)
4. Human (Fight Bite)
5. Black Widow Spider
6. Crotalid
7. Canine
8. Feline
9. Scorpion (Centuroides)

### Appendix D Post-Activity Survey

[Table t8-jetem-9-4-sg13]

**Table t8-jetem-9-4-sg13:**

I am a:	PGY-1	PGY-2	PGY-3	PGY-4	Other: ________
Do you feel that this activity improved your knowledge of the topic?					
		Yes		No	
Would you be interested in doing more activities like these in the future?					
		Yes		No	
Do you have an idea for an in-conference learning activity?					
Comments:					
