# Supplementary material for: A Whodunit Gamified Flipped Classroom For High Yield Bite Injuries And Envenomation
Source: J Educ Teach Emerg Med. 2024 Oct 31;9(4):SG13–23. doi: 10.21980/J88S81 (PMC11537731; doi:10.21980/J88S81)
Supplement: Supplementary file 1 [file 9-4-SG13-Supp1.pptx]

## Slide 1
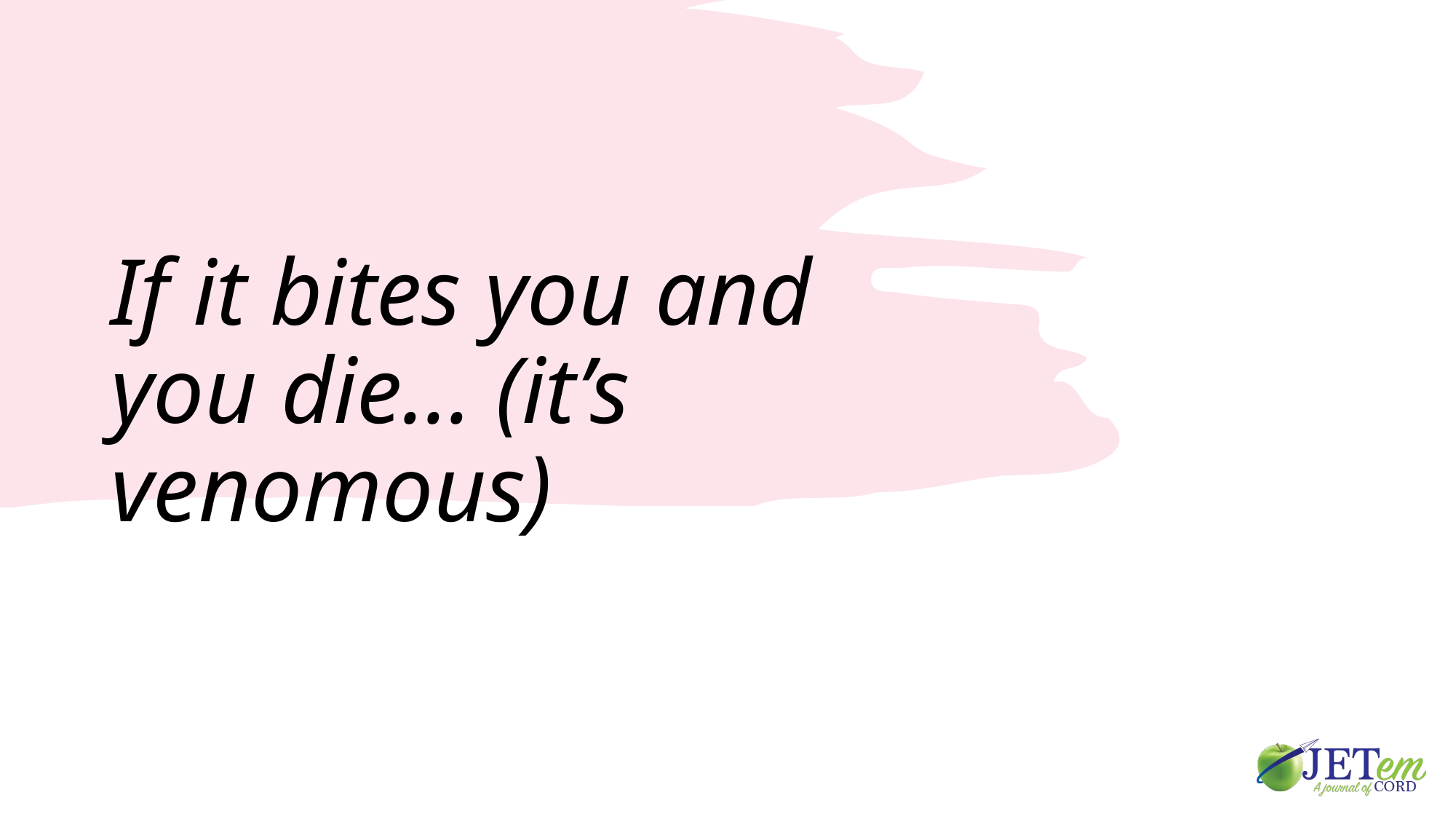

# If it bites you and you die... (it’s venomous)

## Slide 2
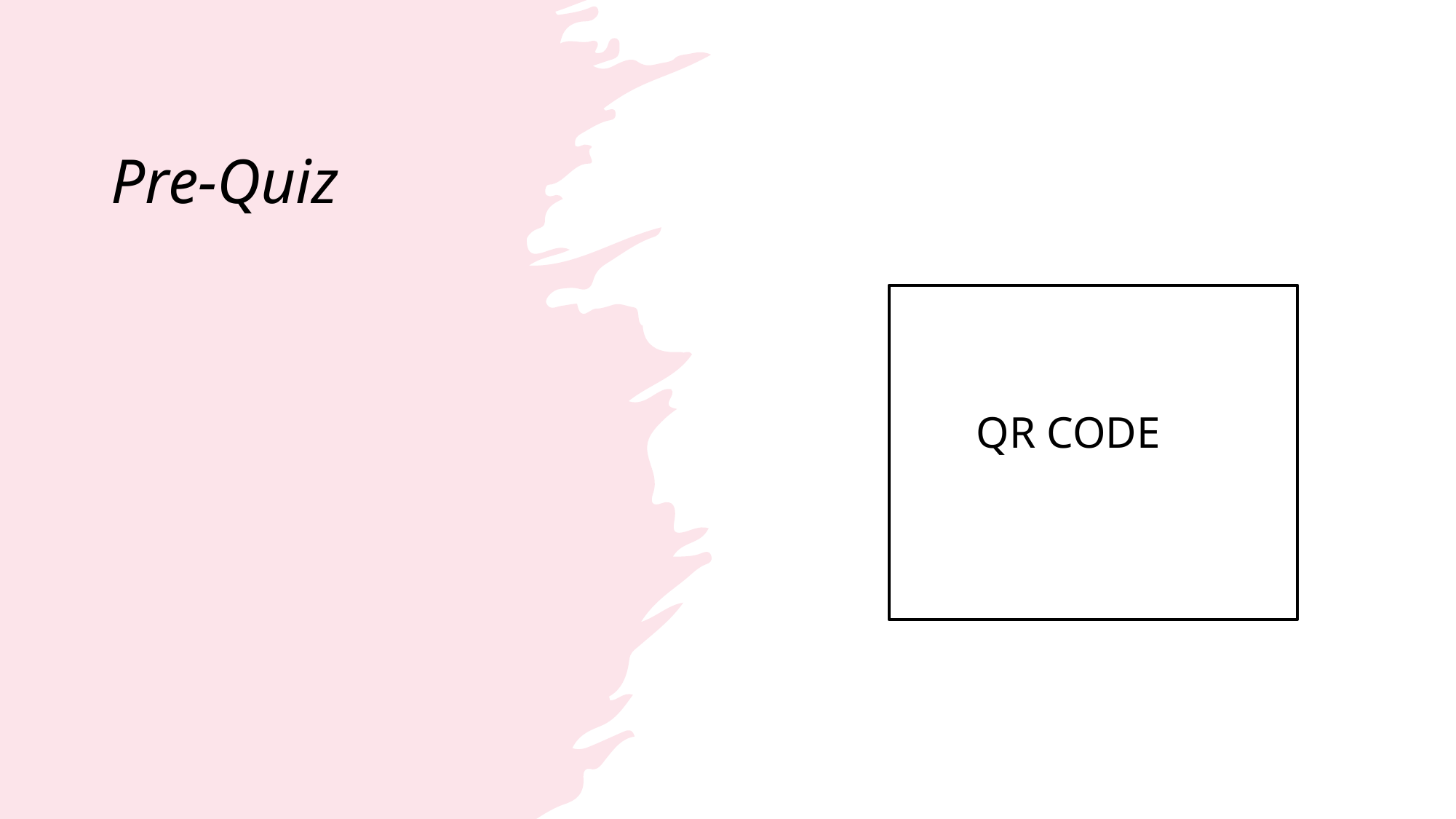

# Pre-Quiz
QR CODE

## Slide 3
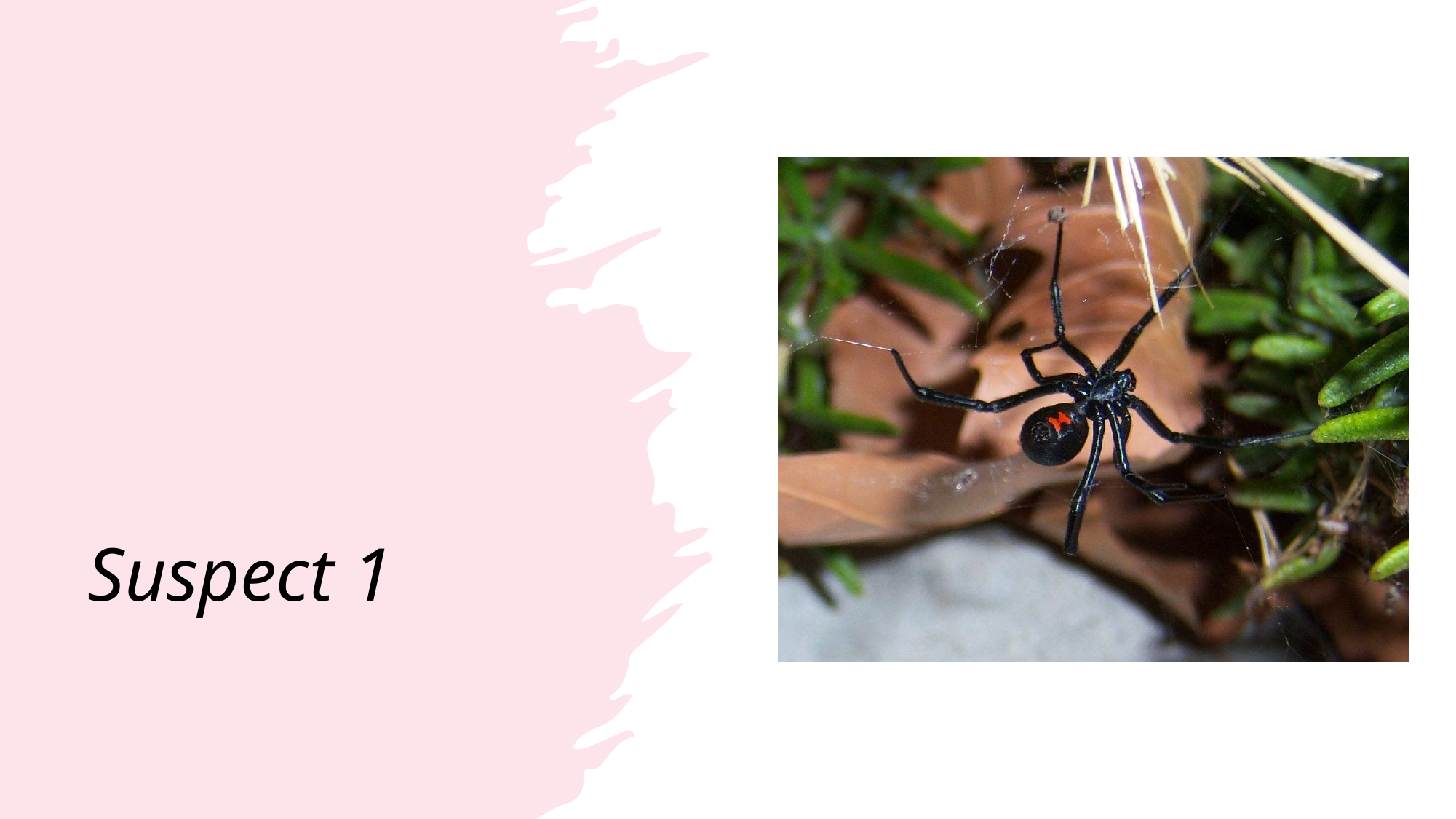

# Suspect 1

## Slide 4
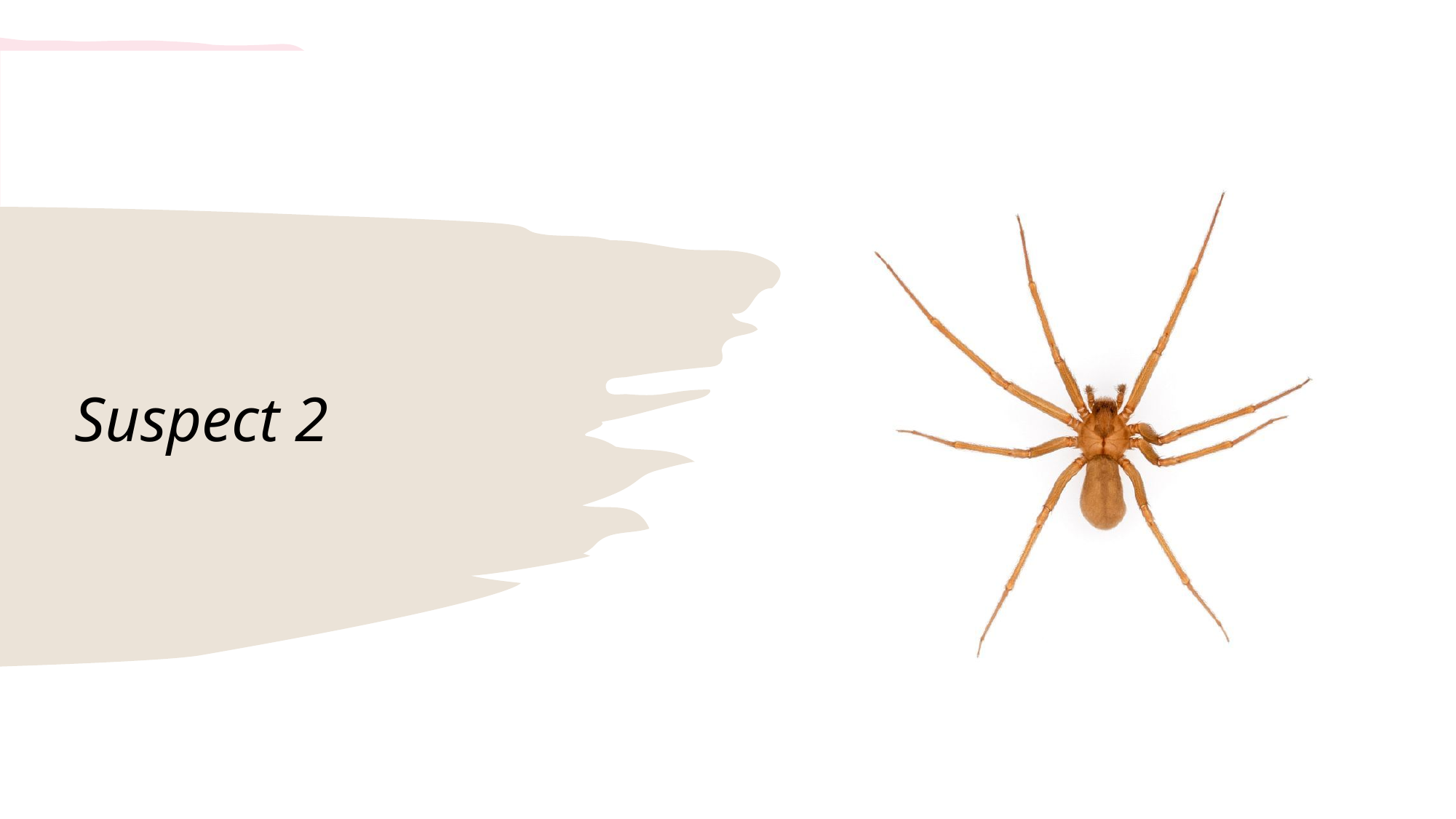

# Suspect 2

## Slide 5
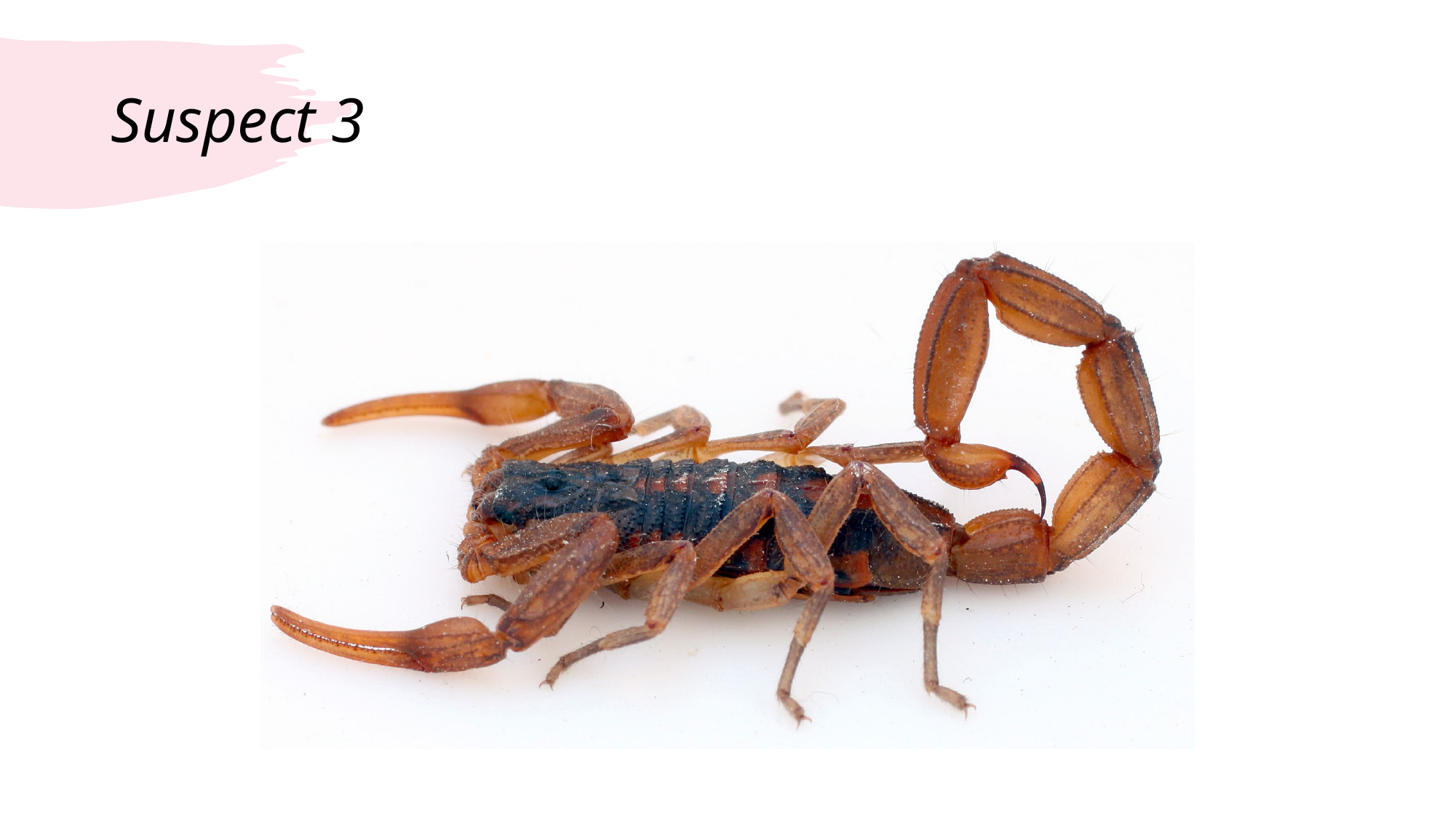

# Suspect 3

## Slide 6
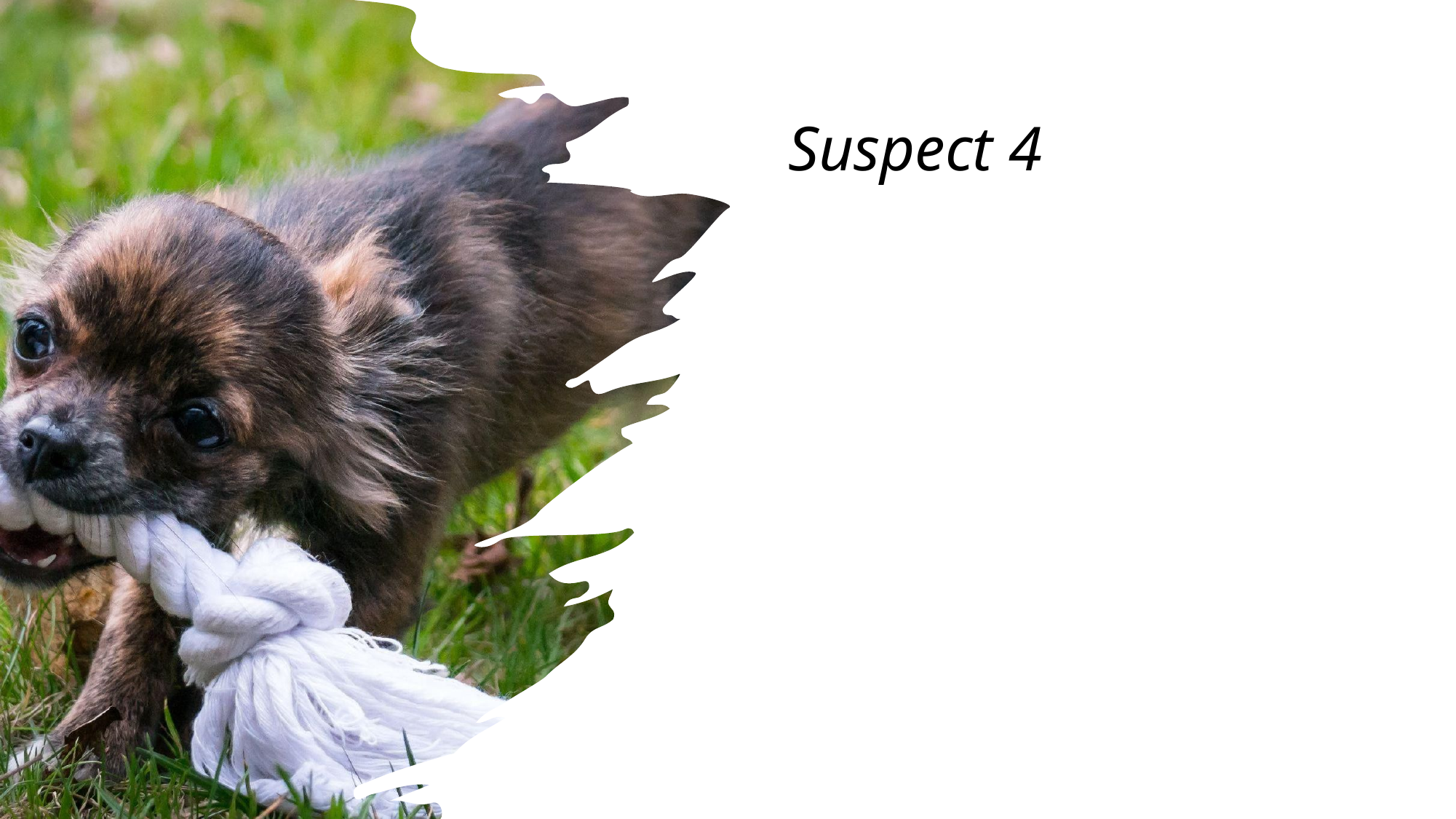

# Suspect 4

## Slide 7
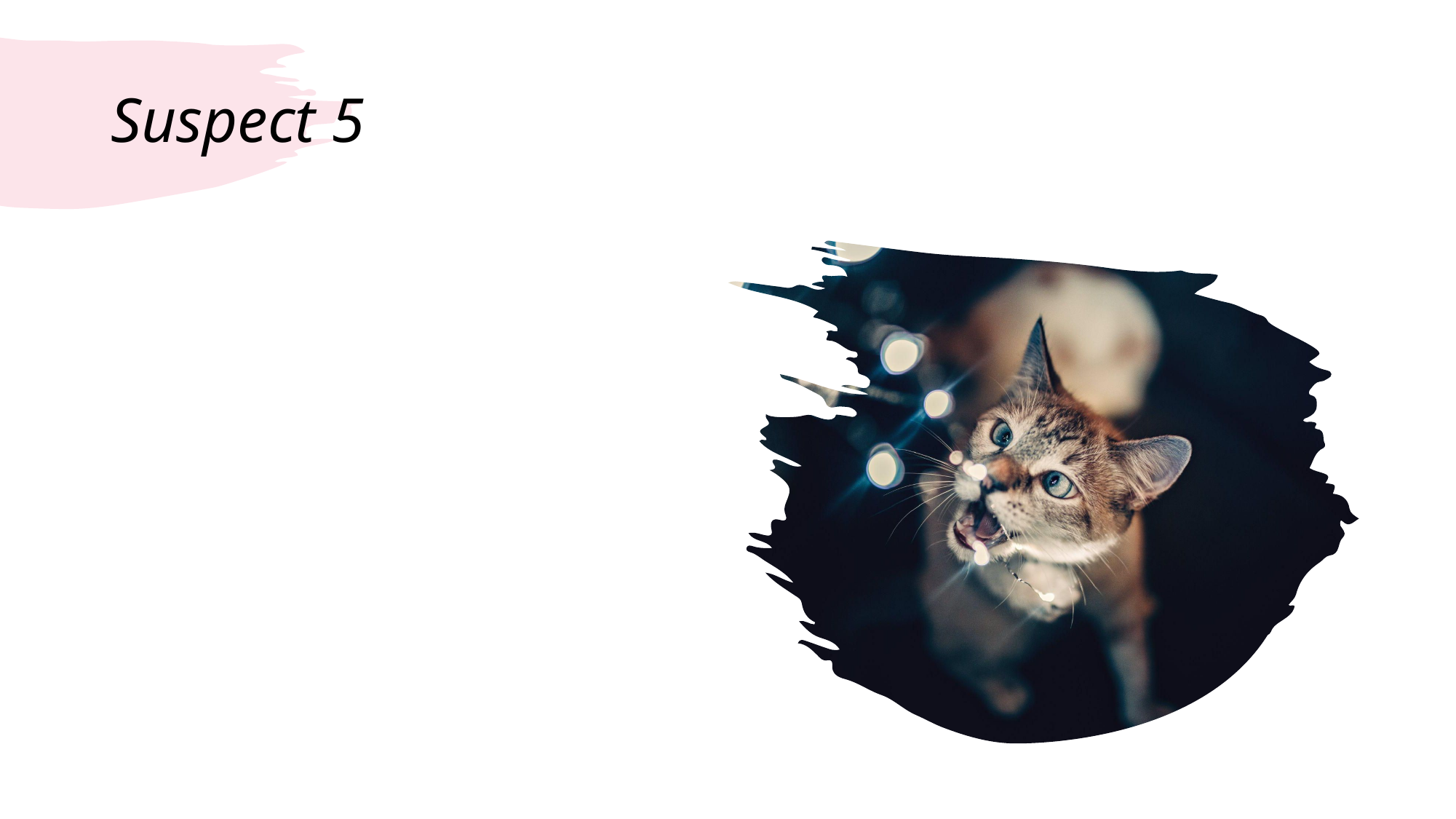

# Suspect 5

## Slide 8
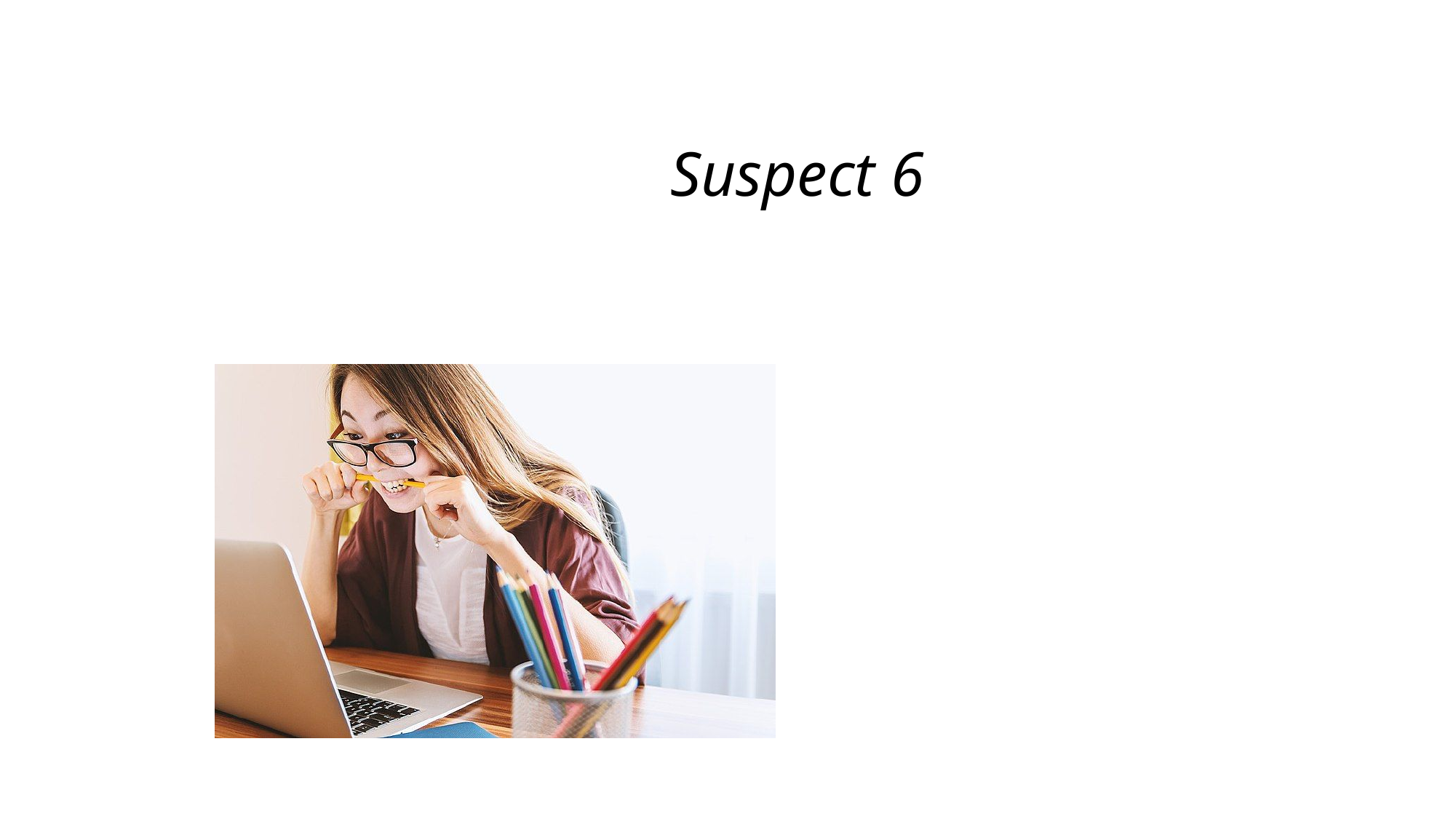

# Suspect 6

## Slide 9
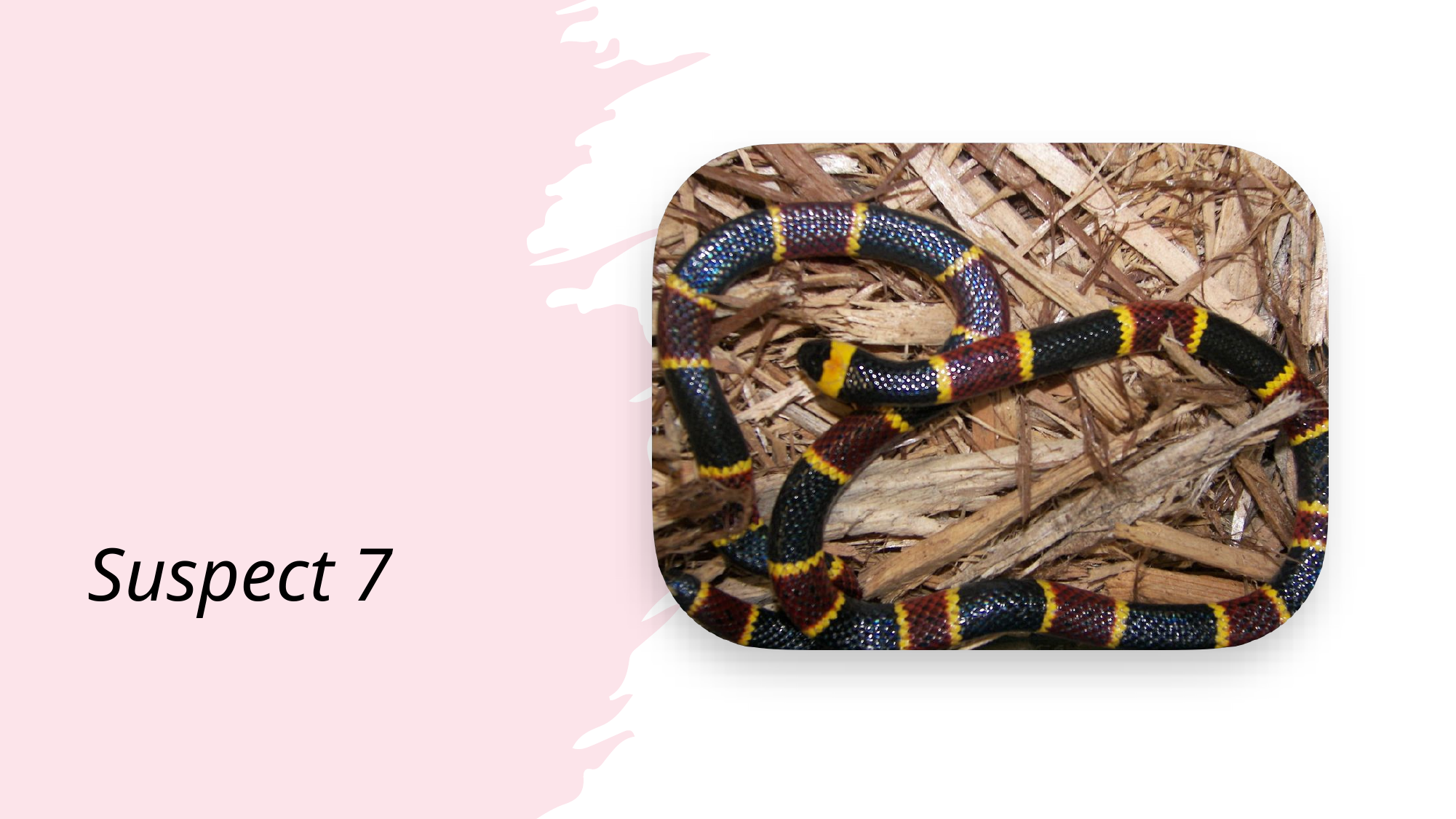

# Suspect 7

## Slide 10
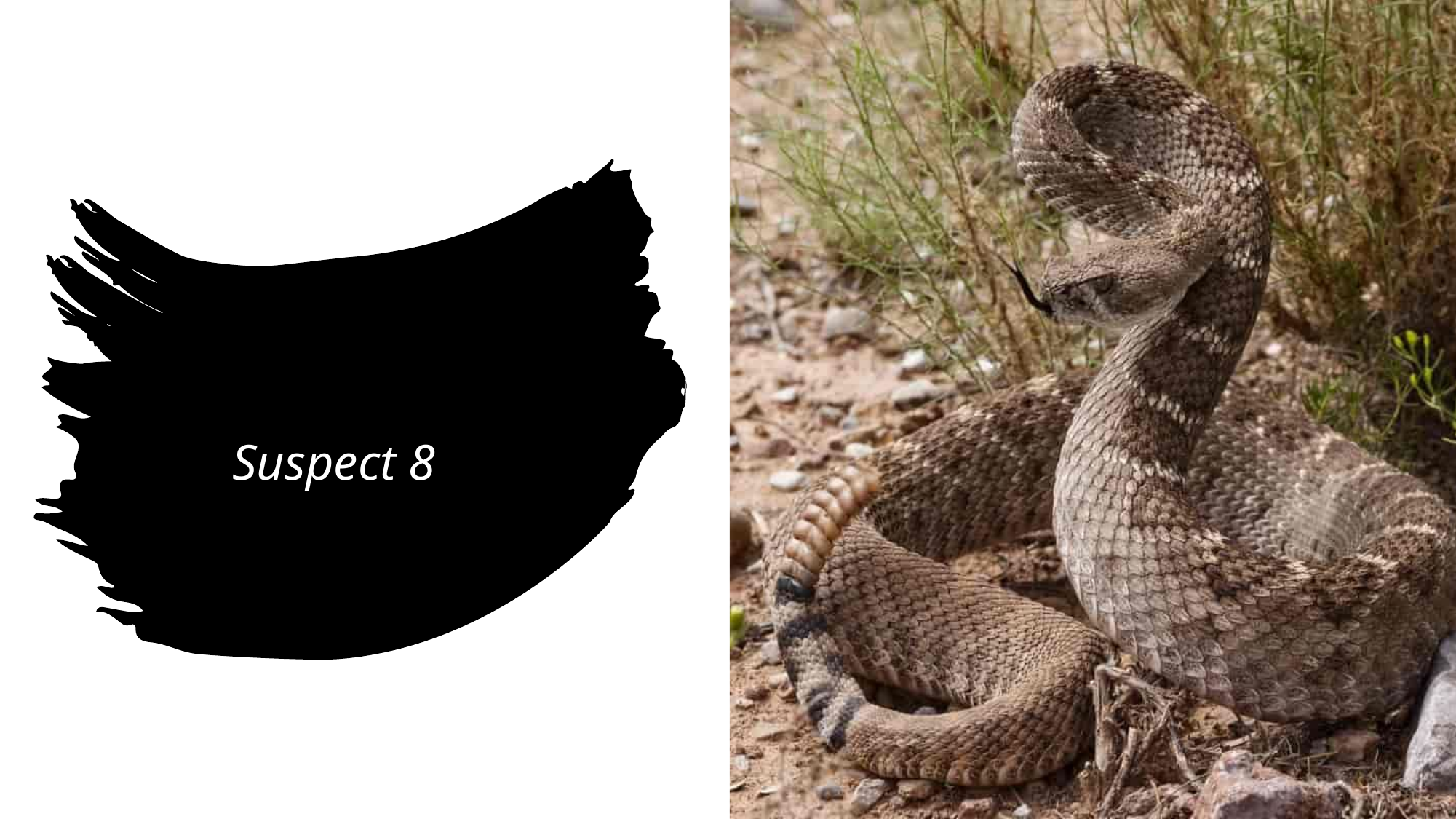

# Suspect 8

## Slide 11
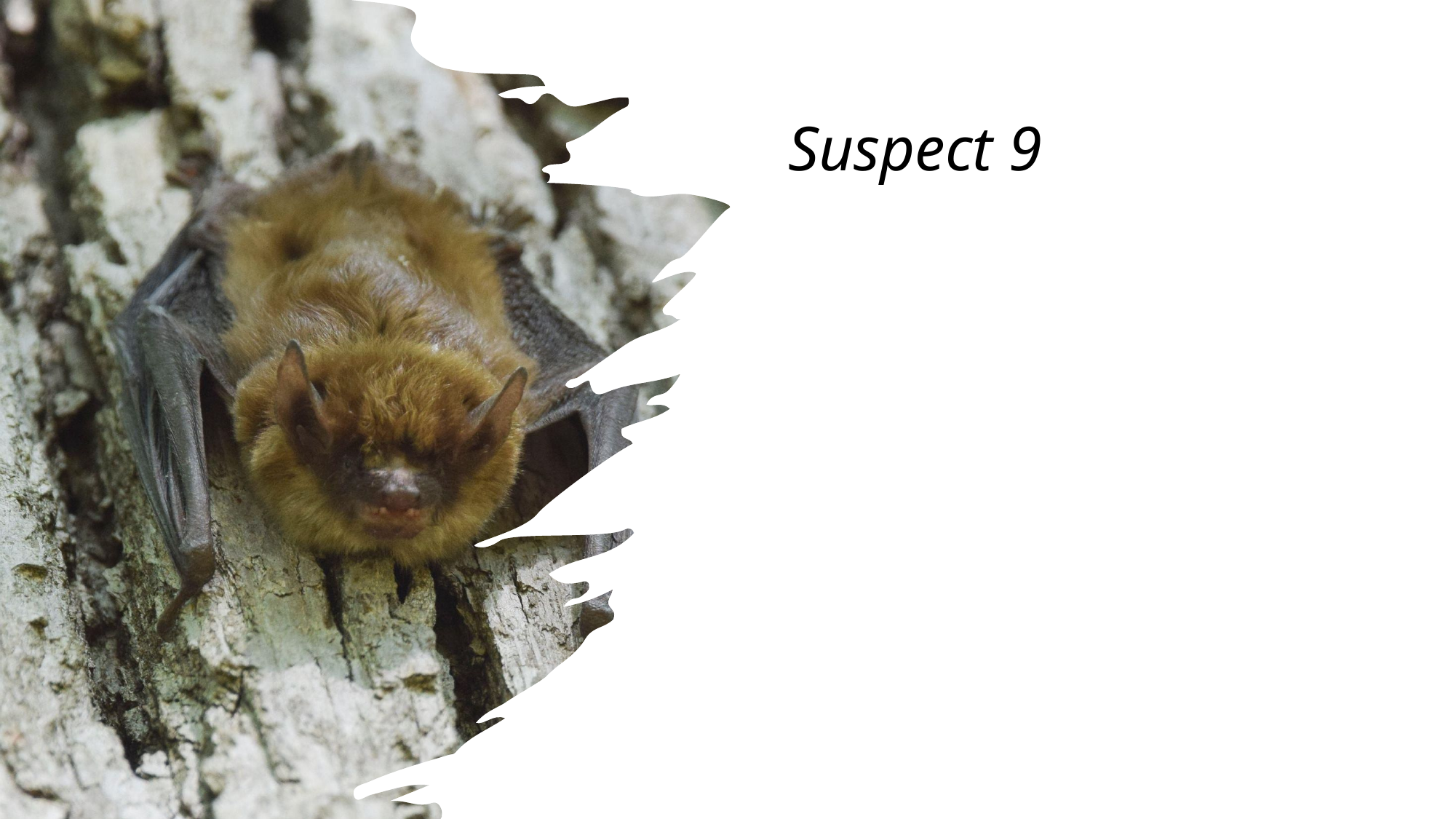

# Suspect 9

## Slide 12
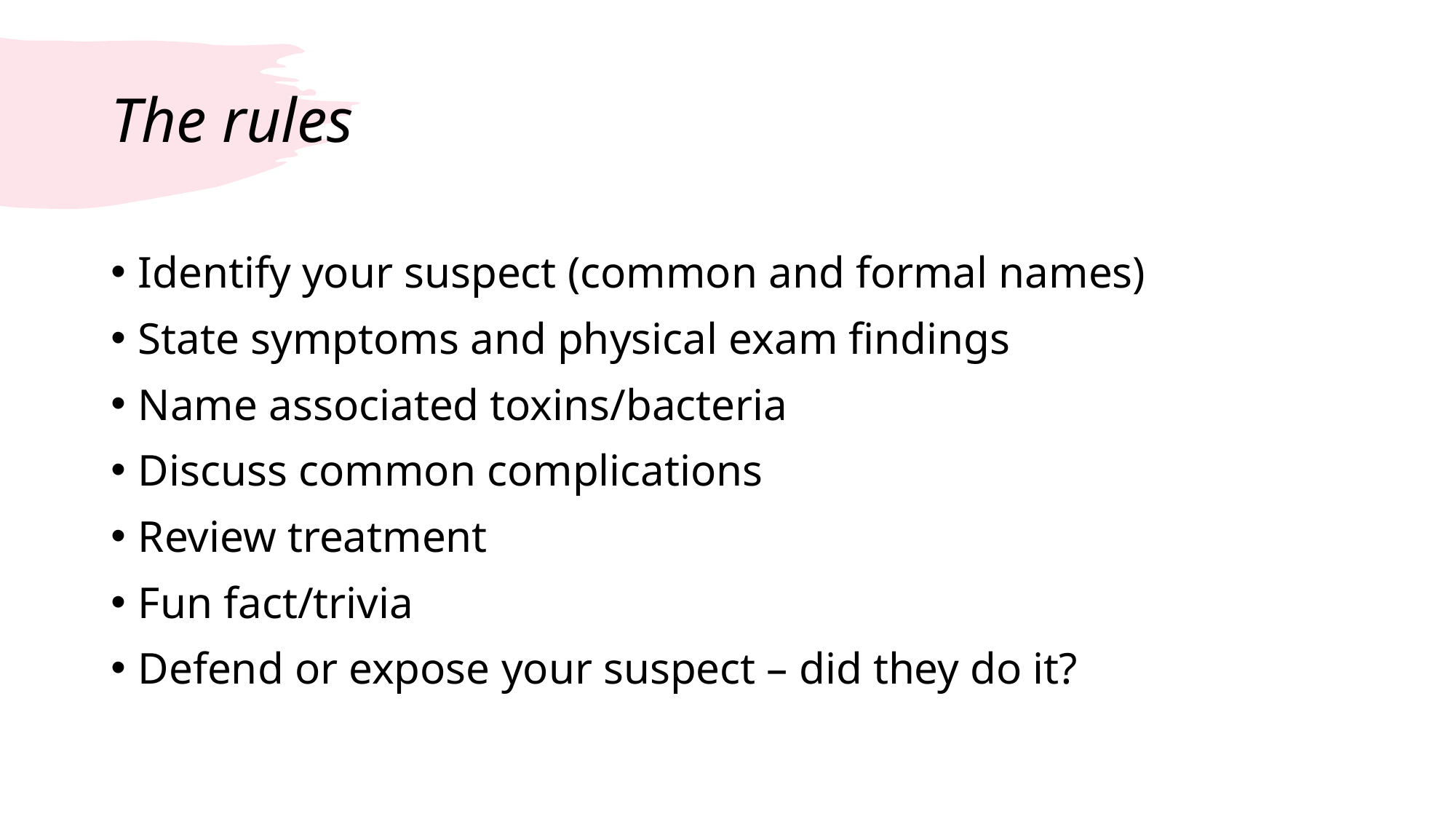

# The rules
Identify your suspect (common and formal names)
State symptoms and physical exam findings
Name associated toxins/bacteria
Discuss common complications
Review treatment
Fun fact/trivia
Defend or expose your suspect – did they do it?

## Slide 13
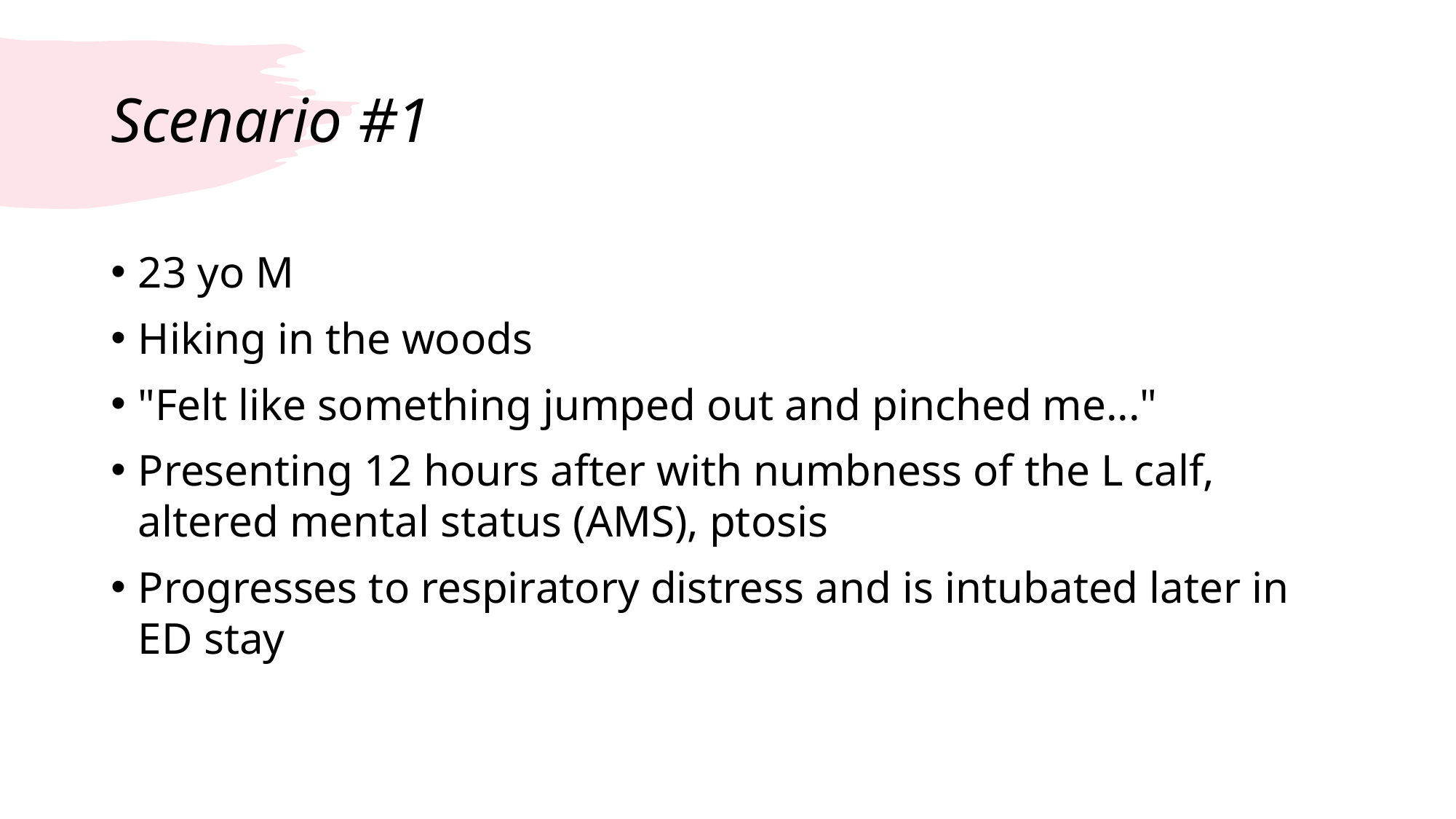

# Scenario #1
23 yo M
Hiking in the woods
"Felt like something jumped out and pinched me..."
Presenting 12 hours after with numbness of the L calf, altered mental status (AMS), ptosis
Progresses to respiratory distress and is intubated later in ED stay

## Slide 14
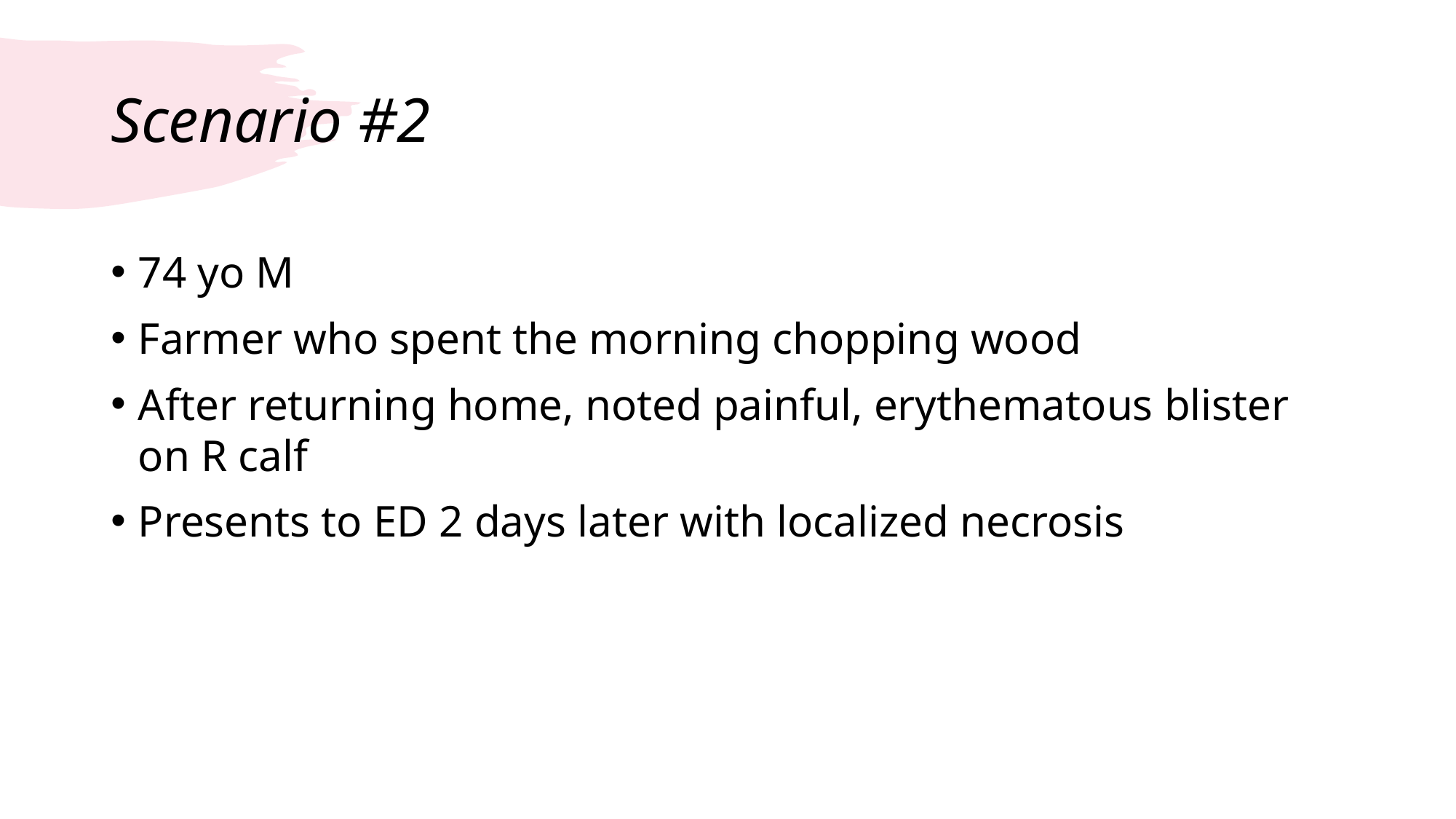

# Scenario #2
74 yo M
Farmer who spent the morning chopping wood
After returning home, noted painful, erythematous blister on R calf
Presents to ED 2 days later with localized necrosis

## Slide 15
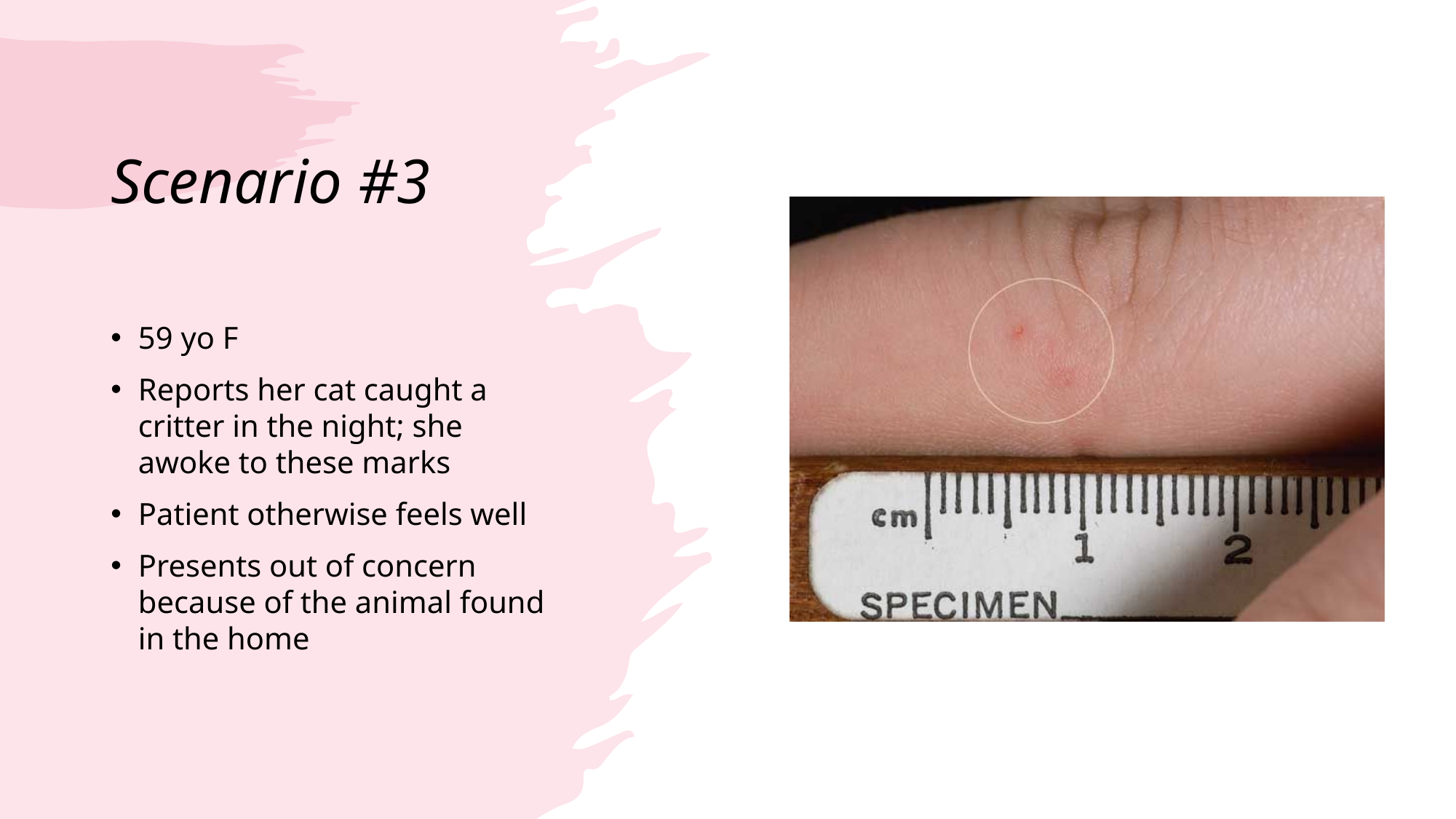

# Scenario #3
59 yo F
Reports her cat caught a critter in the night; she awoke to these marks
Patient otherwise feels well
Presents out of concern because of the animal found in the home

## Slide 16
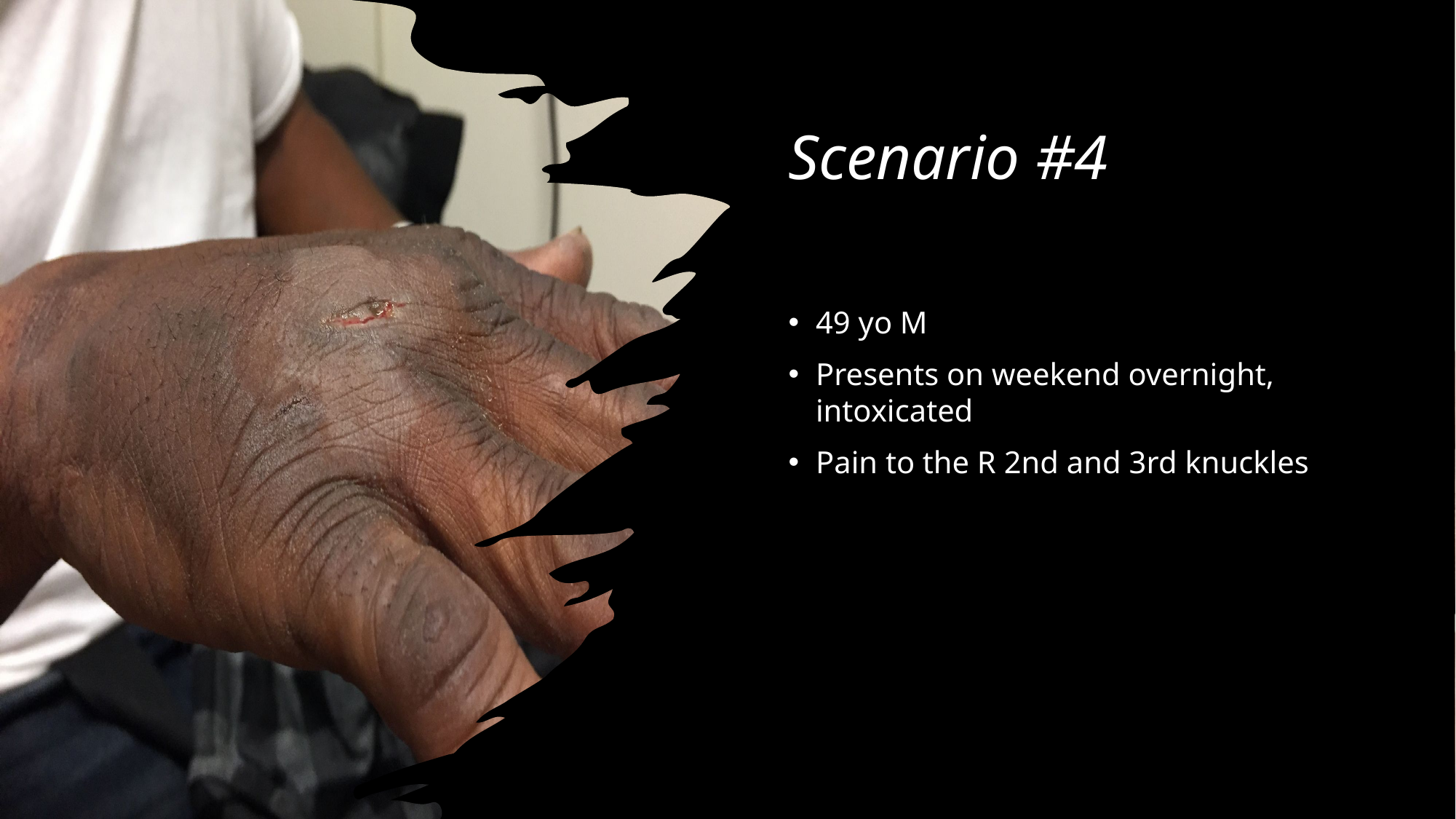

# Scenario #4
49 yo M
Presents on weekend overnight, intoxicated
Pain to the R 2nd and 3rd knuckles

## Slide 17
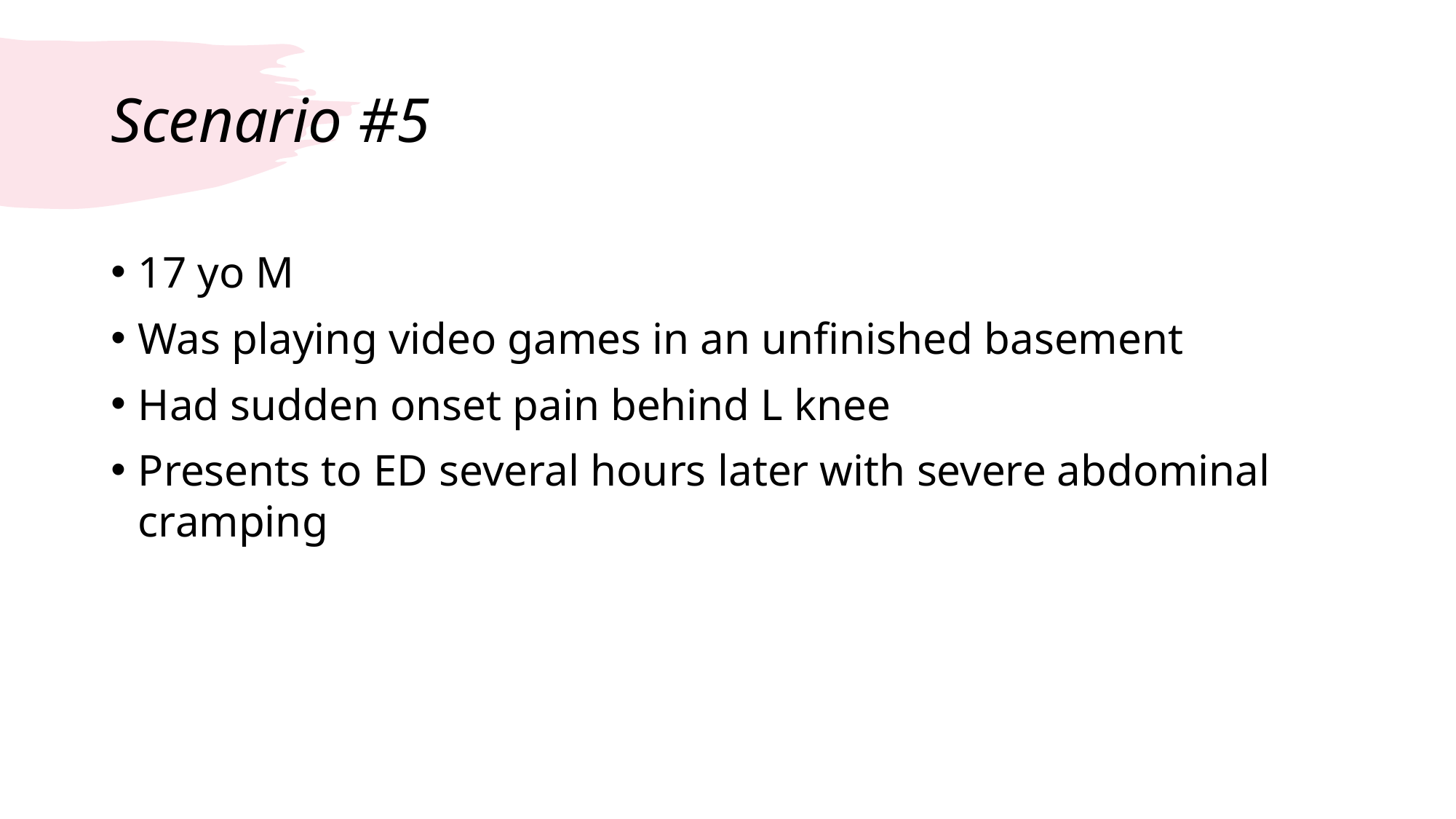

# Scenario #5
17 yo M
Was playing video games in an unfinished basement
Had sudden onset pain behind L knee
Presents to ED several hours later with severe abdominal cramping

## Slide 18
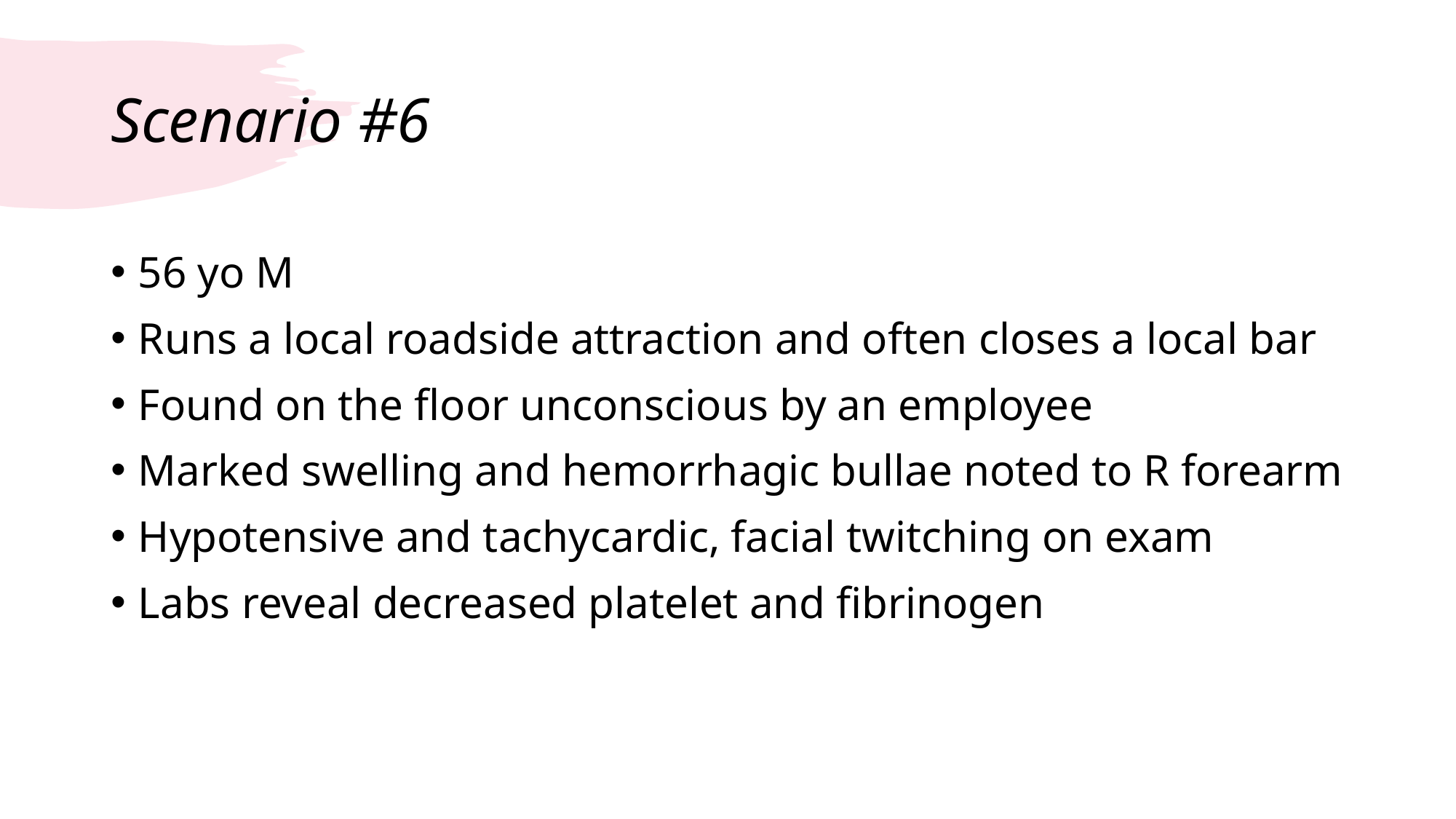

# Scenario #6
56 yo M
Runs a local roadside attraction and often closes a local bar
Found on the floor unconscious by an employee
Marked swelling and hemorrhagic bullae noted to R forearm
Hypotensive and tachycardic, facial twitching on exam
Labs reveal decreased platelet and fibrinogen

## Slide 19
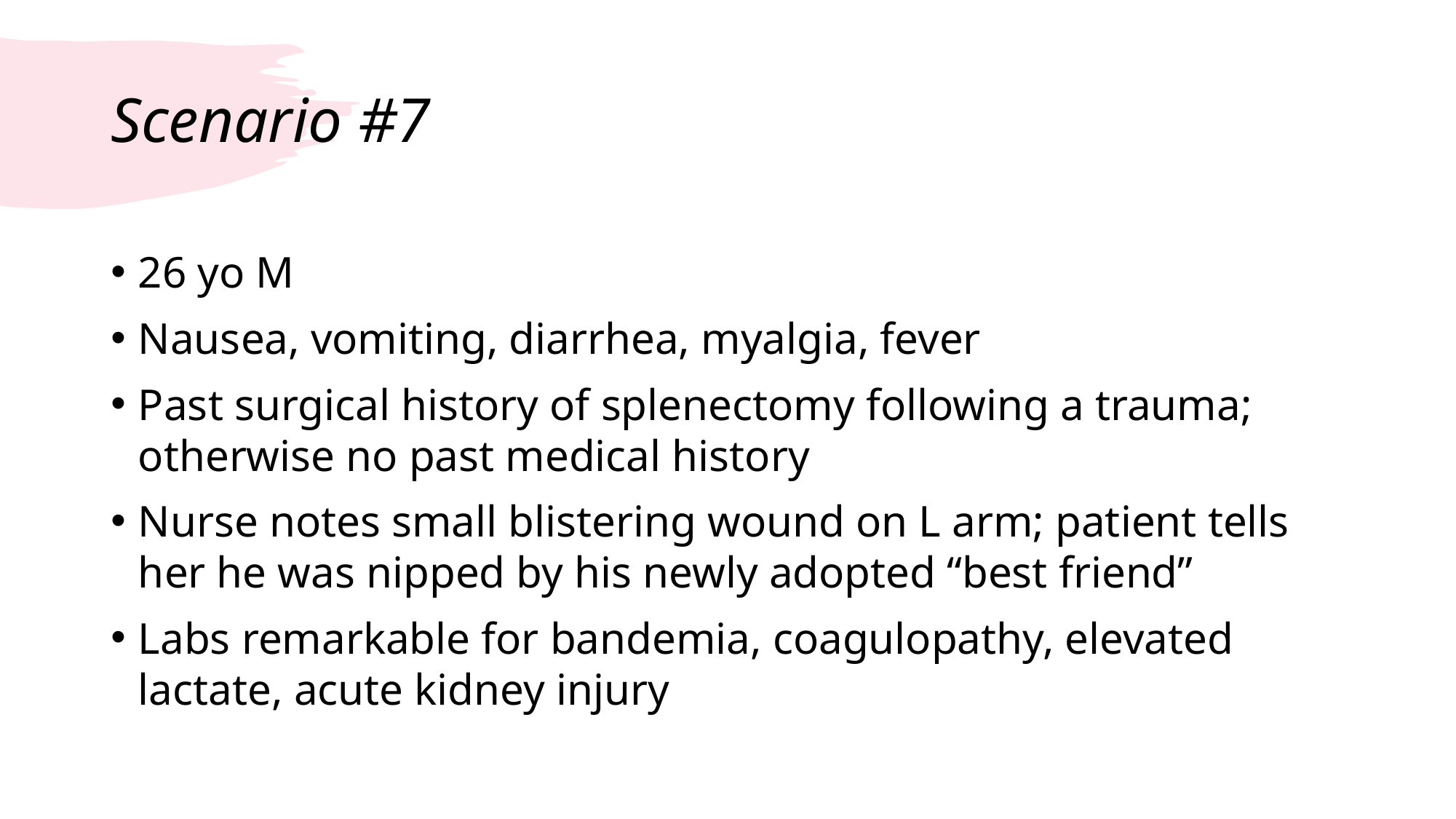

# Scenario #7
26 yo M
Nausea, vomiting, diarrhea, myalgia, fever
Past surgical history of splenectomy following a trauma; otherwise no past medical history
Nurse notes small blistering wound on L arm; patient tells her he was nipped by his newly adopted “best friend”
Labs remarkable for bandemia, coagulopathy, elevated lactate, acute kidney injury

## Slide 20
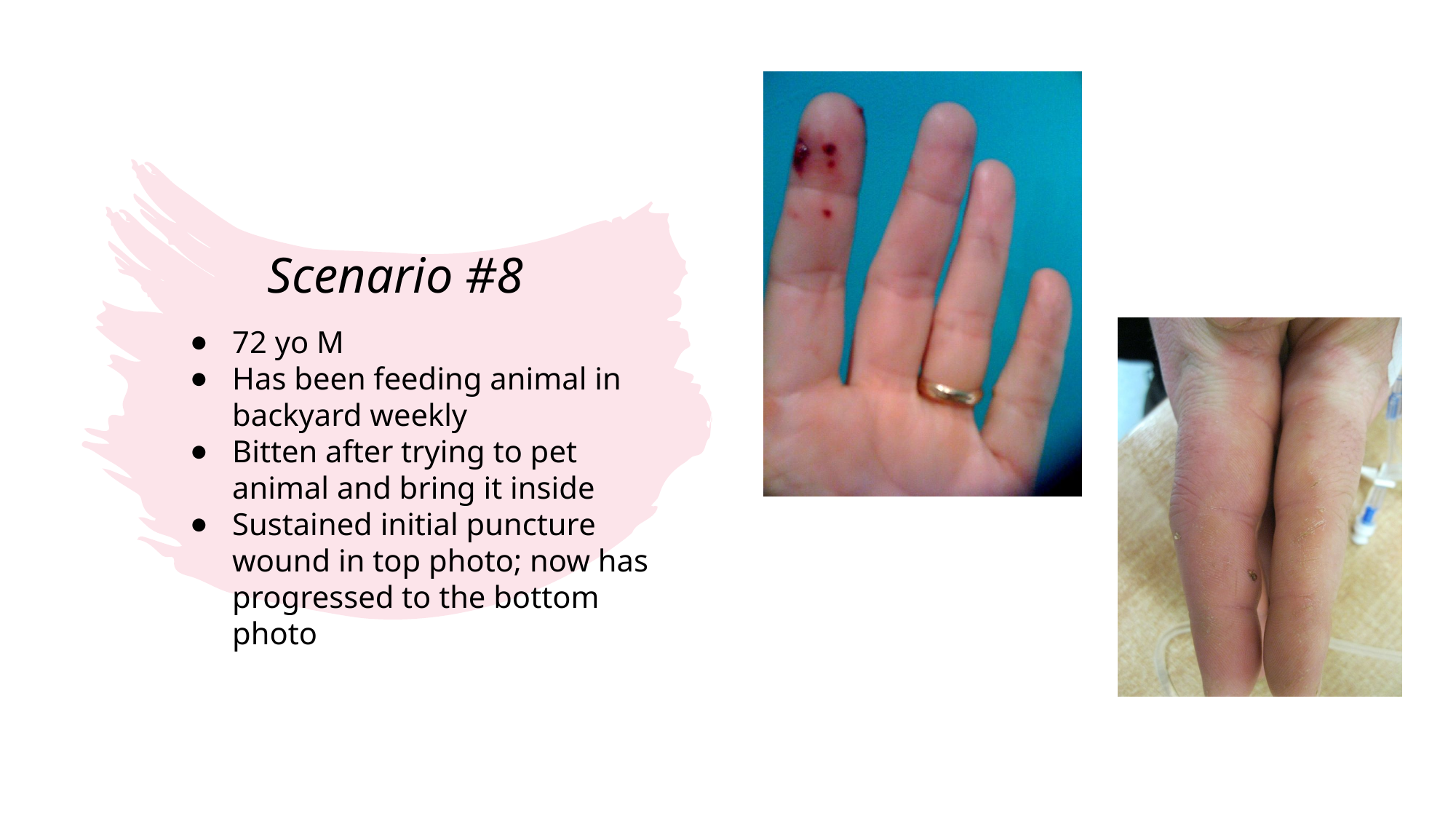

# Scenario #8
72 yo M
Has been feeding animal in backyard weekly
Bitten after trying to pet animal and bring it inside
Sustained initial puncture wound in top photo; now has progressed to the bottom photo

## Slide 21
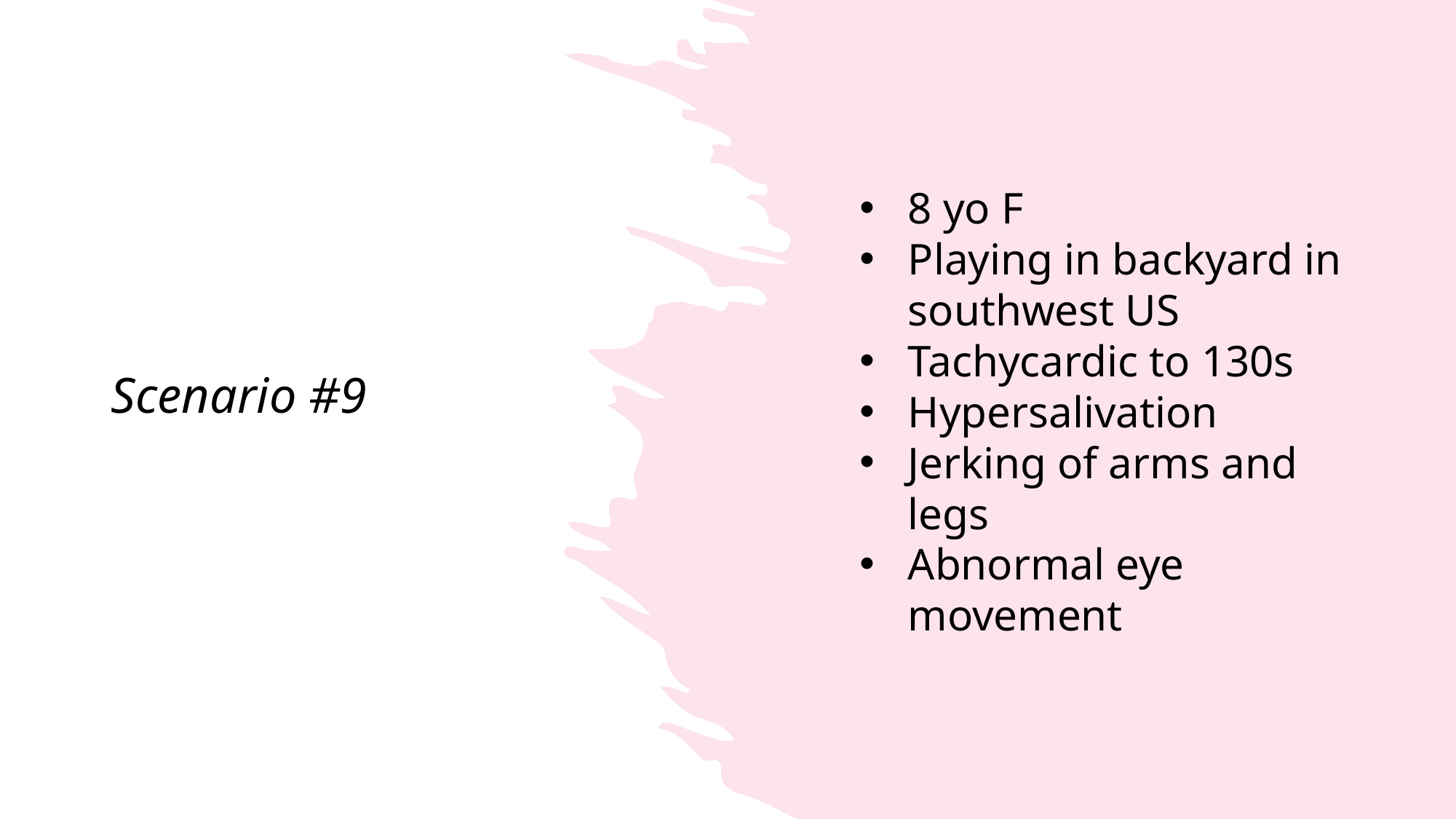

# Scenario #9
8 yo F
Playing in backyard in southwest US
Tachycardic to 130s
Hypersalivation
Jerking of arms and legs
Abnormal eye movement

## Slide 22
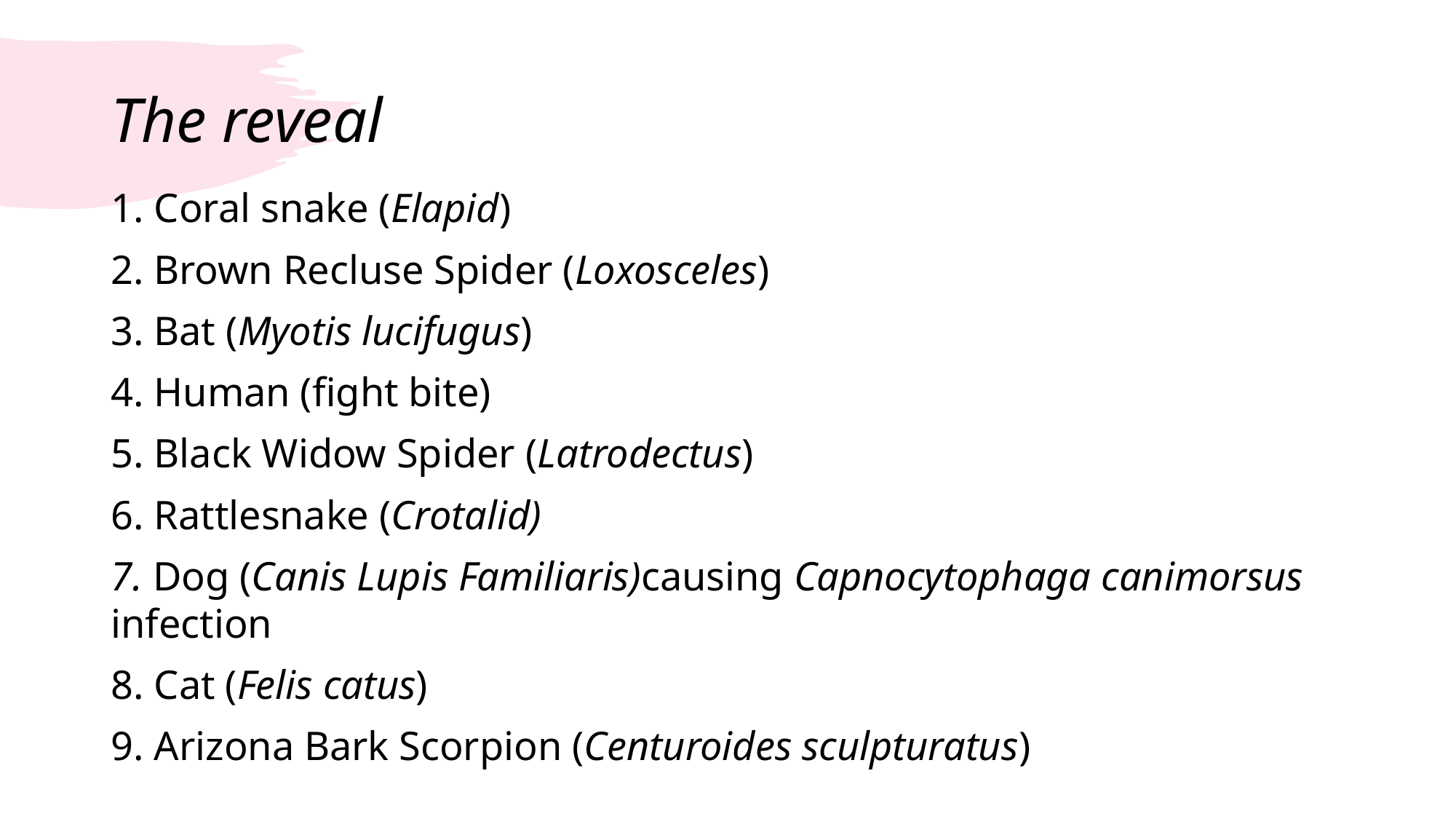

# The reveal
1. Coral snake (Elapid)
2. Brown Recluse Spider (Loxosceles)
3. Bat (Myotis lucifugus)
4. Human (fight bite)
5. Black Widow Spider (Latrodectus)
6. Rattlesnake (Crotalid)
7. Dog (Canis Lupis Familiaris)causing Capnocytophaga canimorsus infection
8. Cat (Felis catus)
9. Arizona Bark Scorpion (Centuroides sculpturatus)

## Slide 23
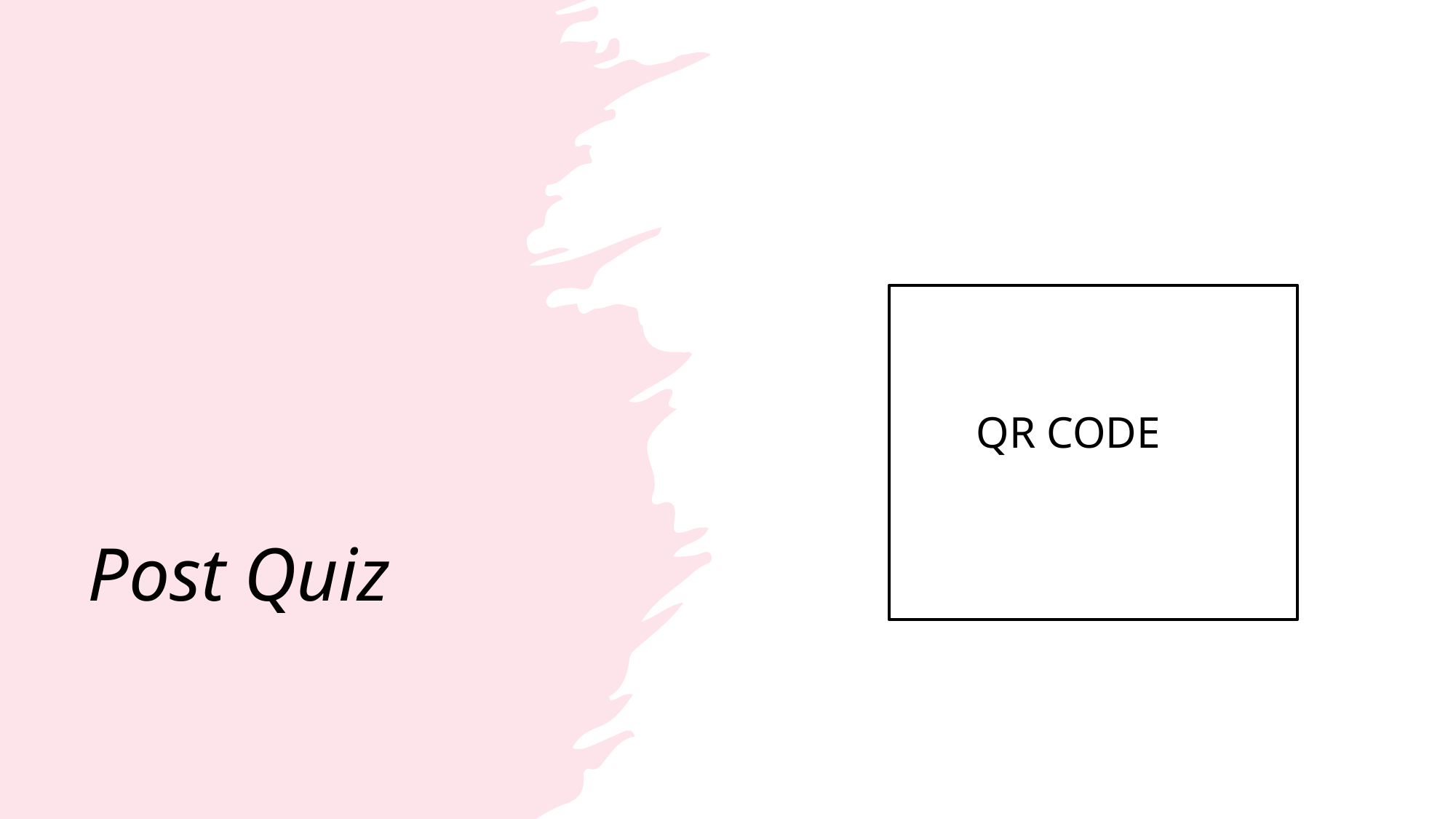

# Post Quiz
QR CODE

## Slide 24
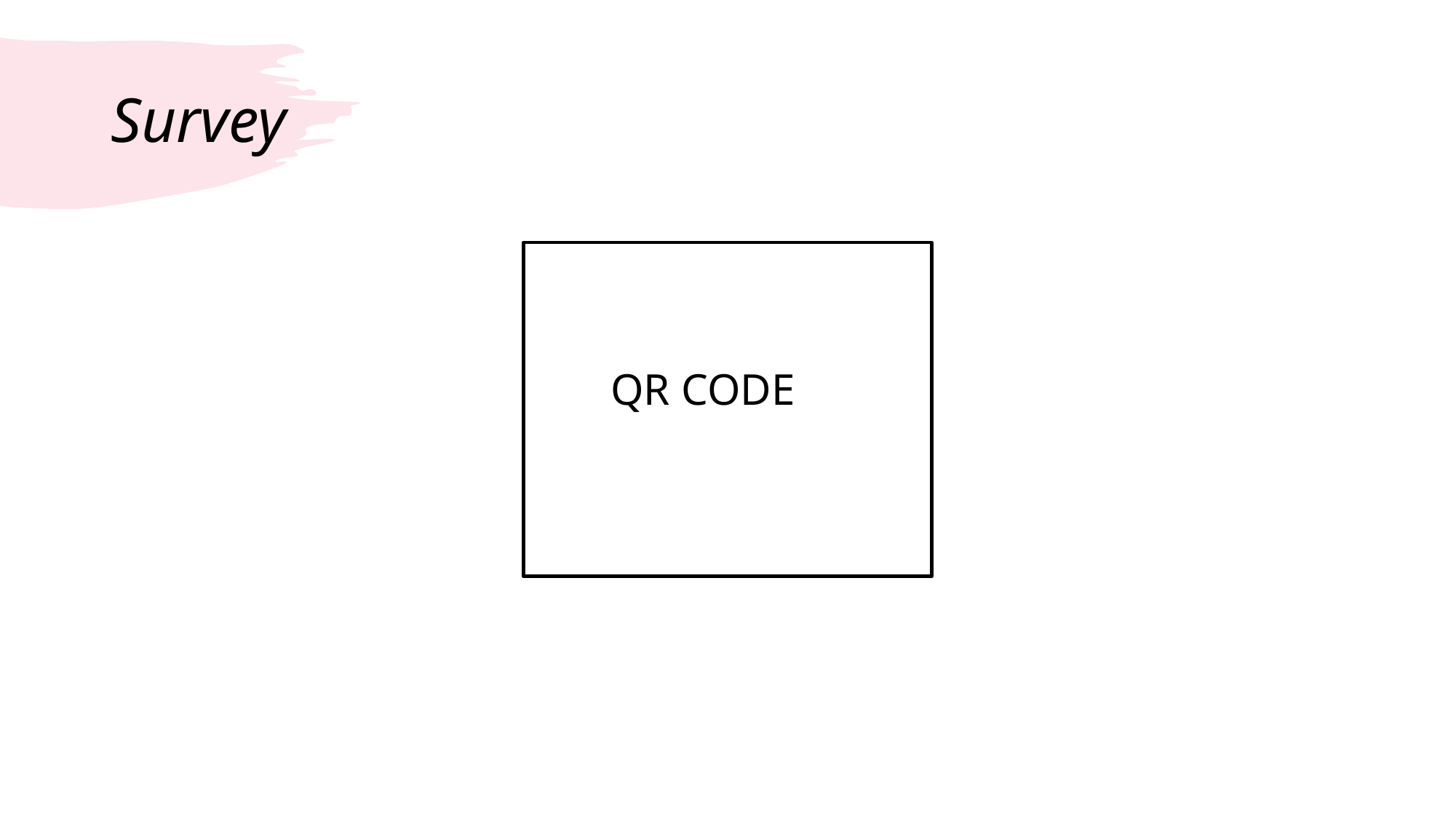

# Survey
QR CODE
